# Comprehensive Genetic Characterization of Four Novel HIV-1 Circulating Recombinant Forms (CRF129_56G, CRF130_A1B, CRF131_A1B, and CRF138_cpx): Insights from Molecular Epidemiology in Cyprus

**DOI:** 10.3390/v16010019

**Published:** 2023-12-21

**Authors:** Cicek Topcu, Vasilis Georgiou, Johana Hezka Rodosthenous, Georgios Siakallis, Elena Katerina Gavala, Christiana Reveka Dimitriou, Evgenia Zeniou, Brian Thomas Foley, Leondios G. Kostrikis

**Affiliations:** 1 Department of Biological Sciences, University of Cyprus, 1 University Avenue, Aglantzia, 2109 Nicosia, Cyprus; 2 AIDS Clinic, Larnaca General Hospital, 6043 Larnaca, Cyprus; 3 T-6 Theoretical Biology and Biophysics, Los Alamos National Laboratory, Los Alamos, NM 87545, USA; 4 Cyprus Academy of Sciences, Letters, and Arts, 60–68 Phaneromenis Street, 1011 Nicosia, Cyprus

**Keywords:** human immunodeficiency virus type 1, circulating recombinant forms, unique recombinant forms, HIV-1 genetic diversity, HIV-1 phylogeny, HIV-1 molecular epidemiology, Cyprus

## Abstract

Molecular investigations of the HIV-1 *pol* region (2253–5250 in the HXB2 genome) were conducted on sequences obtained from 331 individuals infected with HIV-1 in Cyprus between 2017 and 2021. This study unveiled four distinct HIV-1 putative transmission clusters, encompassing 19 previously unidentified HIV-1 recombinants. These recombinants, each comprising eight, three, four, and four sequences, respectively, did not align with previously established Circulating Recombinant Forms (CRFs). To characterize these novel HIV-1 recombinants, near-full-length genome sequences were successfully obtained for 16 of the 19 recombinants (790–8795 in the HXB2 genome) using an in-house-developed RT-PCR assay. Phylogenetic analyses, employing MEGAX and Cluster-Picker, along with confirmatory neighbor-joining tree analyses of subregions, were conducted to identify distinct clusters and determine subtypes. The uniqueness of the HIV-1 recombinants was evident in their exclusive clustering within generated maximum likelihood trees. Recombination analyses highlighted the distinct chimeric nature of these recombinants, with consistent mosaic patterns observed across all sequences within each of the four putative transmission clusters. Conclusive genetic characterization identified four novel HIV-1 CRFs: CRF129_56G, CRF130_A1B, CRF131_A1B, and CRF138_cpx. CRF129_56G exhibited two recombination breakpoints and three fragments of subtypes CRF56_cpx and G. Both CRF130_A1B and CRF131_A1B featured seven recombination breakpoints and eight fragments of subtypes A1 and B. CRF138_cpx displayed five recombination breakpoints and six fragments of subtypes CRF22_01A1 and F2, along with an unclassified fragment. Additional BLAST analyses identified a Unique Recombinant Form (URF) of CRF138_cpx with three additional recombination sites, involving subtype F2, a fragment of unknown subtype origin, and CRF138_cpx. Post-identification, all putative transmission clusters remained active, with CRF130_A1B, CRF131_A1B, and CRF138_cpx clusters exhibiting further growth. Furthermore, international connections were identified through BLAST analyses, linking one sequence from the USA to the CRF130_A1B strain, and three sequences from Belgium and Cameroon to the CRF138_cpx strain. This study contributes valuable insights into the dynamic landscape of HIV-1 diversity and transmission patterns, emphasizing the need for ongoing molecular surveillance and global collaboration in tracking emerging viral variants.

## 1. Introduction

The global HIV-1 epidemic, which emerged in the 1980s, continues to be a profound public health concern. Although considerable strides have been made toward achieving the ambitious 95-95-95 targets set by the Joint United Nations Programme on HIV/AIDS (UNAIDS), the HIV-1 epidemic seems far from ending [1]. The 95-95-95 target was aimed at diagnosing 95% of all individuals living with HIV, supplying antiretroviral therapy (ART) to 95% of all diagnosed individuals, and succeeding viral suppression in 95% of all treated individuals by 2030; however, the most recent global statistics from 2022 revealed figures of 86%, 89%, and 93%, respectively [2]. Nevertheless, the reality remains that global treatment coverage for all those living with HIV-1 can be as low as 65%, with an average of 76%. In the same year, 2022, a global population of 39 million people living with HIV infection, with 1.3 million new infections, was reported [2].

An enduring scientific challenge in the effective treatment and prevention of HIV-1 infection is the virus’s remarkable genetic variability, stemming from rapid viral turnover and a high mutation rate [3,4]. This vast genetic diversity has led to the classification of HIV-1 into four groups (M, N, O, and P), with the major group, M, further divided into ten distinct phylogenetic subtypes (A, B, C, D, F, G, H, J, K, and L) [5,6]. With its exceptional genetic diversity and ongoing global transmission, coupled with its propensity for recombination, HIV-1 frequently gives rise to new recombinant strains [7]. In regions with polyphyletic epidemics, characterized by high genetic diversity, the potential for an individual to be coinfected with multiple distinct HIV-1 strains significantly escalates. This heightened probability of coinfection further enhances the likelihood of new recombinant strain emergence. The dissemination of these recombinant strains leads to the formation of circulating recombinant forms (CRFs). A CRF is defined as an HIV-1 recombinant that is isolated from three or more epidemiologically unconnected individuals infected with HIV-1, provided that these recombinants exhibit an identical intersubtype mosaic structure [8,9]. Presently, there are 140 documented CRFs in the Los Alamos HIV Sequence Database, including two previously identified in Cyprus: CRF04_cpx and CRF91_cpx [10,11,12,13].

Over the course of numerous prospective studies spanning several years, we have conducted extensive investigations into the molecular epidemiology of HIV-1 in Cyprus [13,14,15,16,17,18,19,20]. The dynamic nature of the HIV-1 epidemic in Cyprus has undergone substantial evolution, a phenomenon consistently observed across our comprehensive research endeavors. In our most recent study conducted in Cyprus (2010–2012), a nuanced distribution of HIV-1 strains was unveiled. The predominant circulating strains included subtype B (41.0%), A1 (19.0%), C (7.0%), F1 (8.0%), CRF02_AG (4%), A2 (2.0%), other CRFs (7.0%), and uncharacterized HIV-1 recombinant forms (12%) [15]. The data indicated that 78% of the cohort comprised males, with the remaining 22% being females. Additionally, this study disclosed that 51% of the cohort reported men who have sex with men (MSM) or homo/bisexual contact (HBC) as their route of infection, while 33% reported heterosexual contact (HC). Preliminary insights from an ongoing prospective molecular epidemiology study (2017–2021) (C. Topcu et al., manuscript in preparation for publication) [21] reaffirm the prevalence of polyphyletic infections in Cyprus. Within this evolving landscape, sub-subtype A1 has emerged as the predominant subtype (23.9%), closely followed by subtype B (20.0%), CRF02_AG (14.4%), subtype F1 (4.3%), subtype C (4.3%), subtype G (2.3%), subtype A2 (1%), and other HIV-1 strains (29.8%). The gender distribution was found to align with the most recent published data, reflecting 78.7% males and 22.3% females. Noteworthy in these preliminary findings is that 57.0% reported MSM as their route of infection, while 37.4% reported HC.

Cyprus, located at the crossroads of three continents and characterized by a highly polyphyletic epidemic with diverse HIV-1 strains circulating on the island, provides a fertile ground for the generation of novel CRFs. In this study, we report the identification of four putative transmission clusters of HIV-1 recombinant sequences that were not classified as previously established CRFs. Through amplification and sequencing of the near-full-length HIV-1 genomes of these recombinants, utilizing our in-house RT-PCR assay and conducting comprehensive intersubtype recombination analyses, we have characterized four novel CRFs, CRF129_56G, CRF130_A1B, CRF131_A1B, and CRF138_cpx, all discovered in Cyprus. Additionally, the elucidation of CRF138_cpx led to the identification of a unique recombinant form (URF) of the CRF138_cpx strain. The analyses conducted in this study have provided crucial insights into the nature of these four novel CRFs, predominantly circulating among male individuals, with a strong presence among Cypriot MSM, and later among individuals reporting HBC, many of whom reported contracting the virus in Cyprus. These findings highlight a growing trend in the prevalence of novel CRFs and URFs, particularly in areas characterized by a high degree of HIV-1 genetic diversity. This study also underscores the significance of ongoing prospective molecular epidemiology investigations for the continuous monitoring of HIV-1 transmission, providing a deeper understanding of the intricacies within the HIV-1 epidemic.

## 2. Materials and Methods

### 2.1. Study Participants and Sample Requirements

This study encompassed 18 HIV-1 nucleotide sequences originating from 18 individuals. Sixteen of these sequences were procured during a prospective molecular epidemiology study (C. Topcu et al., manuscript in preparation for publication) [21] involving 305 participants, conducted from 9 March 2017 to 14 October 2021. The remaining two sequences were obtained through routine HIV-1 genotypic drug resistance analyses carried out by our laboratory during the same timeframe. The Cyprus National Bioethics Committee (CNBC) granted approval for all experimental procedures conducted in this study. Bioethical approval was granted for two distinct periods: 9 March 2017 to 13 October 2019 (approval number EEBK EΠ 2017.01.23, approval date 20 February 2017) and 14 October 2019 to 14 October 2021 (approval number EEBK ΕΠ 2019 71, approval date 2 September 2019). The inclusion criteria required that all individuals participating in the prospective molecular epidemiology study (C. Topcu et al., manuscript in preparation for publication) [21] be consenting individuals who were either newly diagnosed with HIV-1 infection or chronically infected and had not received antiretroviral therapy (antiretroviral-naïve). Henceforth, the individuals participating in this study, who were sampled in the aforementioned prospective molecular epidemiology study (C. Topcu et al., manuscript in preparation for publication) [21], met these inclusion criteria, while the remaining two individuals who were sampled as part of the routine HIV-1 genotypic drug resistance analyses were reported to have previously received antiretroviral therapy.

The collection of data from the prospective molecular epidemiology study (C. Topcu et al., manuscript in preparation for publication) [21] followed the guidelines and regulations set by the CNBC and the Office of the Commissioner for Personal Data Protection in Cyprus. Accordingly, written informed consent was obtained from all study participants. Qualified medical personnel administered a questionnaire that captured detailed clinical, epidemiological, behavioral, and demographic information of the study participants. Furthermore, to maintain the confidentiality of the study participants, a double coding method was implemented, thus ensuring the anonymity and privacy of the study participants. The samples utilized in this study were assigned the following laboratory identification numbers: CY397, CY413, CY448, CY512, CY525, CY526, CY529, CY537, CY584, CY590, CY620, CY625, CY639, CY697, CY710, CY805, CY824, and CY842.

The blood samples, consent forms, and questionnaires belonging to the study participants were collected at the Grigorios HIV Clinic of Larnaca General Hospital. This collection process strictly adhered to the guidelines and regulations outlined by the CNBC. The Grigorios HIV Clinic is the sole national clinical facility in Cyprus responsible for the comprehensive care of individuals infected with HIV-1. Therefore, it presents a unique and valuable opportunity to establish a cohort of individuals who accurately represent the HIV-1 epidemic in Cyprus.

### 2.2. Plasma Isolation and HIV-1 RNA Extraction

Blood samples were collected at the Grigorios HIV Clinic of Larnaca General Hospital and then transported to the Laboratory of Biotechnology and Molecular Virology at the University of Cyprus for further processing of plasma and peripheral blood mononuclear cell (PBMC) isolation. The isolation from whole blood was performed within two hours of sample collection, in accordance with the previously defined methodology [19]. Later, HIV-1 RNA was extracted from plasma according to a previously published protocol [20].

### 2.3. PCR Amplification and Sanger Sequencing of the HIV-1 Pol Region

All blood samples collected from 9 March 2017 to 14 October 2021 as part of the routine HIV-1 genotypic drug resistance analyses were subjected to RT-PCR amplification and sequencing of the HIV-1* pol* (*protease*, *reverse transcriptase*, *integrase*, and partial *vif*) region (2253–5350 in the HXB2 genome) using a previously established touchdown HIV-1 pol RT–PCR assay [13,19,20]. The HIV-1 genotypic subtypes were determined using REGA HIV-1 subtyping tool, version 3.0 (REGA 3.0) [22]. Then, the identification of drug resistance mutations was carried out using the HIVdb Program of the Stanford University HIV Drug Resistance Database [23,24]. Following the exclusion of samples from nonconsenting individuals and subsequent samples from the same individual (returning individual), 331 HIV-1 *pol* region sequences from 331 individuals were obtained. An amount of 305 of the 331 sequences belonged to individuals who met the inclusion criteria of the aforementioned prospective molecular epidemiology study (C. Topcu et al., manuscript in preparation for publication) [21] and hence were included in its cohort.

### 2.4. Phylogenetic Analyses of HIV-1 Pol Region Sequences

Afterward, a comprehensive phylogenetic analysis was performed on the 331 HIV-1 *pol* region (2253–5250 in the HXB2 genome) nucleotide sequences utilizing formerly defined bioinformatics tools and parameters [13]. This analysis was executed as part of our routine monthly phylogenetic analyses, which were performed for a surveillance monitoring system, in an effort to reveal the ever-evolving HIV-1 transmission dynamics in Cyprus in near real time. Briefly, this involved the use of Molecular Evolutionary Genetics Analysis (MEGA X) software for multiple sequence alignment using the ClustalW algorithm, followed by the construction of a maximum likelihood phylogenetic tree based on the general time-reversible (GTR) nucleotide substitution model with a gamma distribution and 1000 bootstrap replicates [25,26,27]. Cluster Picker software (version 1.2.3) was then employed to facilitate phylogenetic clustering analysis, with predefined criteria requiring a genetic distance threshold of 0.045 and a minimum of 70% bootstrap support for clustering [14,28]. FigTree v1.4.3 software was later used to visualize and edit the resulting maximum likelihood phylogenetic tree [29]. Phylogenetic clusters were designated as putative transmission clusters, and this classification criterion mandated a minimum of three samples clustering together, adhering to established criteria and methodologies [14]. Within this analysis, among others, four putative transmission clusters were identified, comprising a total of 19 HIV-1 recombinant sequences. Notably, these 19 HIV-1 sequences represented four separate unknown HIV-1 recombinant strains as previously defined by their HIV-1 genotypic subtypes. The determined HIV-1 genotypic subtypes were distinct from previously established CRFs, suggesting the potential emergence of four distinct novel CRF strains within the *pol* region of the virus.

### 2.5. PCR Amplification and Sanger Sequencing of the Near-Full-Length HIV-1 Genome

To potentially identify newly emerging CRFs, this study aimed to obtain near-full-length HIV-1 genome (790–8795 in the HXB2 genome) sequences that would be utilized in further detailed analyses of intersubtype recombination breakpoints. This was achieved through an in-house-developed near-full-length HIV-1 genome RT–PCR assay, which was previously explained in detail [13,20]. In brief, the assay encompasses three overlapping amplicons that collectively cover the entire HIV-1 genome. These amplicons are generated through the utilization of established PCR assays, specifically the formerly described HIV-1 pol RT–PCR assay (2253–5250 in the HXB2 genome) [19], the HIV-1 gag RT–PCR assay (790–2292 in the HXB2 genome) [20], and the HIV-1 env RT–PCR assay (5041–8795 in the HXB2 genome) [20]. All three PCR assays were tailored with HIV-1-specific primers for both PCR amplification and sequencing, targeting a range of HIV-1 group M subtypes, CRFs, and recombinant strains. As such, near-full-length HIV-1 genomes, which comprised *gag*, *pol*, *vif*, *vpr*, *tat*, *rev*, *vpu*, *env*, and partial *nef* regions, were successfully amplified and sequenced for 16 of the 19 HIV-1 recombinants. Unfortunately, two of the HIV-1 recombinants could not be successfully sequenced in the HIV-1 *env* region, and one in the HIV-1 *gag* region due to the presence of low-quality reads. Consequently, one of the samples for which sequencing of the HIV-1 *env* region failed was excluded from this study, whereas the remaining two samples were included as partial sequences. This failure in sequencing could be attributable to the high prevalence of secondary HIV-1 strains generated by frameshift mutations.

### 2.6. Phylogenetic Analyses of Near-Full-Length HIV-1 Genome Sequences

Subsequently, the 16 near-full-length HIV-1 genome nucleotide sequences and 2 partial sequences obtained were utilized to conduct a repetition of the phylogenetic analyses. For each of the four groups of HIV-1 recombinant query sequences, a separate multiple sequence alignment was constructed with a reference dataset of all known HIV-1 group M subtypes (A, B, C, D, F, G, H, J, K, and L) and CRFs. These multiple sequence alignments were created using RIP Alignment 2020, which was accessed from the Los Alamos HIV Sequence Database (http://www.hiv.lanl.gov (accessed on 13 November 2023)). The aforementioned methodology was then reapplied to perform phylogenetic analyses with the aim of demonstrating the distinct and exclusive phylogenetic clustering of these four separate groups of HIV-1 recombinant query sequences, thereby providing evidence of their uniqueness in the context of HIV-1 genetic diversity. Following this, the HIV-1 genotypic subtypes based on the near-full-length HIV-1 genome sequences were determined using the REGA HIV-1 Subtyping Tool Version 3.0 [7].

### 2.7. Intersubtype Recombination Analyses

To verify the presence of potentially novel recombination patterns, thorough intersubtype recombination breakpoint analyses were conducted. Specifically, bootscan and similarity plot analyses were executed on each of the 18 HIV-1 recombinant query sequences. These analyses were conducted utilizing formerly defined bioinformatics tools and parameters [13]. Specifically, MEGA X software was employed for the construction of the multiple sequence alignments of the query sequences and the reference dataset using the Clustal W algorithm [25,26]. Then, AliView software was used to visualize and manually edit the multiple sequence alignment [30]. Briefly, these analyses were executed using reference datasets consisting of pure HIV-1 group M subtypes (A, B, C, D, F, G, H, J, K, and L), CRF22_01A1, and CRF56_cpx, all of which were sourced from the Los Alamos HIV Sequence Database (http://www.hiv.lanl.gov (accessed on 13 November 2023)). To enhance the reference datasets, additional sequences were incorporated through Basic Local Alignment Search Tool (BLAST) analyses with the top BLAST hits for subtypes B, F2, CRF22_01A1, and CRF56_cpx. To facilitate robust intersubtype recombination analyses and enhance sequence comparisons, reference sequences were selected based on their tight clustering with the study samples observed in the maximum likelihood trees and corroborated by the outcomes of the BLAST analyses. The HIV BLAST tool available from the Los Alamos HIV Sequence Database, which can be accessed at https://www.hiv.lanl.gov/content/sequence/BASIC_BLAST/basic_blast.html (accessed on 13 November 2023) was employed to execute BLAST analyses, retaining the default parameters. Accession numbers of the sequences included in each reference dataset as well as the accession numbers of the query sequences are explicitly listed in the “Data Availability Statement” as well as in the figure legends.

SimPlot v3.5.1 software was used to identify and assess putative intersubtype recombination breakpoints through bootscan and similarity plot analyses [31]. These analyses were conducted with parameters adopted from a previous study [17], which included a sliding window of 400 nucleotides overlapping by 40 nucleotides and the generation of 1000 bootstrap replicates. Confirmation of the defined putative intersubtype recombination breakpoints was carried out using a jumping profile hidden Markov model (jpHMM) [32] and the Recombinant Identification Program (RIP) [33].

To further confirm the intersubtype recombination breakpoints and subtype origin of each fragment, a phylogenetic-based approach was employed. Accordingly, confirmatory neighbor-joining tree analyses of subregions were performed using MEGA X software by utilizing the abovementioned reference datasets [25]. Maximum likelihood trees were also generated; however, their inclusion did not yield significant impacts on the observed results. Given the substantial number of trees that needed construction, a more time-efficient approach was adopted, favoring the neighbor-joining method for its expediency. In the phylogenetic analyses, the Kimura two-parameter nucleotide substitution model was used along with 1000 bootstrap replicates to evaluate the trustworthiness of the phylogenetic clustering outcomes [34]. For every fragment within each HIV-1 recombinant query sequence, a neighbor-joining phylogenetic tree was created. A bootstrap support value of 70% was regarded as conclusive in determining the subtype origin, following the previously published threshold [13]. To provide a summary of intersubtype mosaicism, the Recombinant HIV-1 Drawing Tool (https://www.hiv.lanl.gov/content/sequence/DRAW_CRF/recom_mapper.html (accessed on 13 November 2023)) was employed to create four distinct mosaic genomic maps representing the genetic composition of the 18 HIV-1 recombinants.

As a result, to investigate other sequences that might have connections with the query sequences and exhibit similar intersubtype mosaicism, BLAST analyses were conducted using the HIV BLAST tool (https://www.hiv.lanl.gov/content/sequence/BASIC_BLAST/basic_blast.html (accessed on 13 November 2023)). In this analysis, the set of 18 HIV-1 recombinant sequences was employed as the query dataset, where each sequence was subjected to these comparative phylogenetic analyses individually following formerly published procedures [13]. In short, sequences were analyzed as both near-full-length genome sequences and partial sequences that were selected according to the readily accessible regions of the HIV-1 genome in the database. After identifying other sequences that potentially have connections with the query sequences, thorough intersubtype recombination breakpoint analyses were repeated in SimPlot v3.5.1 and MEGA X software for bootscan and similarity plot analyses and confirmatory neighbor-joining tree analyses of subregions, respectively, following the abovementioned procedures [25,31].

## 3. Results

### 3.1. Clinical, Epidemiological, Behavioral, and Demographic Information of the Study Participants

Eighteen consenting individuals infected with HIV-1 participated in this study. All study participants were living in Cyprus during the sampling period, and their blood samples were collected between 9 March 2017 and 14 October 2021. Table 1 displays a summary of the detailed clinical, epidemiological, behavioral, and demographic information of the study cohort. All study participants were male (n = 18, 100%). Their ages varied between 30 and 61 years, with the most frequently represented age group being “30–39” (n = 8, 44.4%). The majority of study participants were from Cyprus (n = 14, 77.8%), with a smaller number of participants coming from Lebanon (n = 1, 5.6%), Greece (n = 1, 5.6%), Cameroon (n = 1, 5.6%), and Romania (n = 1, 5.6%). Similarly, the study participants reported being infected with HIV-1 predominantly in Cyprus (n = 14, 77.8%), with fewer participants reporting Greece (n = 2, 11.1%), Venezuela (n = 1, 5.6%) and Romania (n = 1, 5.6%) as their country of infection. It should be underscored that the information pertaining to the country of infection was self-reported by the study participants, introducing a potential for unreliability in this data. Among the study participants, the most prevalent route of infection was recorded as MSM (n = 9, 50.0%), with HBC (n = 5, 27.8%) and HC (n = 3, 16.7%) being the other reported routes of infection. It is noteworthy that MSM refers to individuals who exclusively engage in sexual activities with men, while HBC pertains to those who have sexual contacts with both genders. Additionally, one of the individuals reported belonging to both the injecting drug user (IDU) and MSM groups (n = 1, 5.6%). Most of the individuals who participated in this study resided in Nicosia, the capital of the island (n = 12, 66.7%), at the time of sampling, while the remainder of the study participants resided in Larnaca (n = 5, 27.8%) and Paralimni (n = 1, 56%).

The blood samples obtained from the individuals enrolled in this study were examined, revealing HIV-1 viral RNA loads that ranged from approximately 300 to 9,000,000 RNA copies/mL of plasma. Of the 18 individuals, 14 were newly diagnosed with HIV-1 infection and had never received antiretroviral therapy (n = 14, 77.8%). The remaining four individuals were chronically infected with HIV-1 (n = 4, 22.2%), two of whom reported to have received antiretroviral therapy prior to sampling (n = 2, 11.1%). Furthermore, the drug resistance analysis of the obtained HIV-1 *pol* region nucleotide sequences revealed the presence of four major drug resistance mutations and 23 accessory drug resistance mutations linked to nonnucleoside reverse transcriptase inhibitors (NNRTIs), protease inhibitors (PIs), and integrase strand transfer inhibitors (INSTIs). Specifically, the determined drug resistance mutations included E138A (n = 3) associated with NNRTIs; L10I (n = 7), K20I/R (n = 7), L33F (n = 1), and A71V (n = 5) associated with PIs; and L74I (n = 4) associated with INSTIs. Consistent with the results of the mutational analysis, the majority of the HIV-1 recombinant sequences were identified to be susceptible to the antiretroviral drugs assessed by the HIVdb Program [23,24]. However, three HIV-1 recombinant sequences, which contained the E138A major drug resistance mutation, exhibited potential low-level resistance to etravirine (ETR) and low-level resistance to rilpivirine (RPV). Table 2 presents a summary of the clinical status, antiretroviral treatment status, and drug resistance mutations of the study participants in relation to the recombinant putative transmission cluster they joined.

### 3.2. Phylogenetic Analyses and HIV-1 Genotypic Subtypes

**Figure 1:**
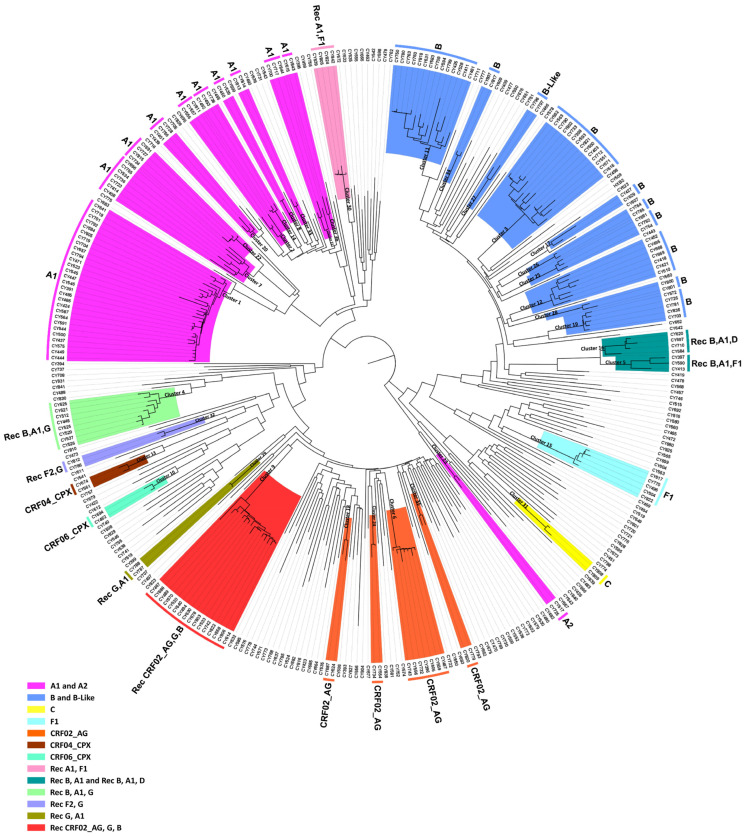
Latest monthly maximum likelihood phylogenetic tree dating to September 2021. The maximum likelihood tree was constructed for the monthly phylogenetic analyses performed as part of the near-real-time surveillance monitoring system. This system was implemented by our Laboratory of Biotechnology and Molecular Virology and was applied to our laboratory website to deliver up-to-date visualization analytics with regards to the near-real-time phylogenetic clustering of individuals infected with HIV-1. The maximum likelihood tree was constructed using the HIV-1 pol region (2253–5250 in the HXB2 genome) nucleotide sequences isolated from whole blood samples of individuals infected with HIV-1 collected in Cyprus from 9 March 2017 to 14 October 2021. The cohort used for the construction of the maximum likelihood phylogenetic tree included individuals that were either newly diagnosed or chronically infected with HIV-1. Additionally, the cohort included individuals without consideration of their combination antiretroviral therapy (cART) status. MEGA X software was employed for the construction of the maximum likelihood tree. Phylogenetic clusters were categorized as putative transmission clusters if minimum of three sequences were clustered together. The identified phylogenetic clusters are color coded according to the determined HIV-1 genotypic subtypes, which are indicated at the periphery of each phylogenetic cluster. The color coding is also described on the bottom left of the figure.

From 9 March 2017 to 14 October 2021, a total of 455 blood samples were collected for the purpose of routine HIV-1 genotypic drug resistance analyses. Following the removal of PCR-negative samples and exclusion of samples from the nonconsenting and returning individuals, a final cohort of 331 HIV-1 *pol* region (2253–5250 in the HXB2 genome) nucleotide sequences was retained as the focus of our investigation. Figure 1 shows the monthly phylogenetic tree of September 2021, which includes these 331 samples. Within this analysis, a total of 36 phylogenetic molecular clusters were identified, 19 of which were putative transmission clusters. An amount of 4 out of the 19 putative transmission clusters comprised 19 HIV-1 recombinant sequences, which represented four separate unknown HIV-1 recombinant strains as previously defined by their HIV-1 genotypic subtypes. The HIV-1 genotypic subtypes were identified as belonging to HIV-1 recombinant strains that differed from the previously established CRFs, indicating the possibility of four new and unique CRF strains emerging within the virus’s *pol* region. Notably, the fifth HIV-1 recombinant putative transmission cluster comprising 16 HIV-1 recombinant sequences was previously identified and characterized as a novel CRF, CRF91_cpx, in Cyprus [13].

The HIV-1 recombinant putative transmission clusters had eight, three, four, and four HIV-1 recombinant query sequences. The largest HIV-1 recombinant putative transmission cluster comprised eight samples. The HIV-1 genotypic subtypes of these sequences were defined as “Rec. of B, A1, G”. The second HIV-1 recombinant putative transmission cluster comprised three samples. The HIV-1 genotypic subtypes of these sequences were defined either as “Rec. of B, A1” or “Rec. of B, A1, F1”. The third HIV-1 recombinant putative transmission cluster comprised four samples. The HIV-1 genotypic subtypes of these sequences were defined either as “Rec. of B, A1” or as “Rec. of B, A1, D”. Finally, the fourth HIV-1 recombinant putative transmission cluster comprised four samples. The HIV-1 genotypic subtypes of these sequences were defined as “Rec. of A1, F1”. Moreover, the HIV-1 genotypic subtypes that were reanalyzed based on the near-full-length genome sequences also supported the sequences as belonging to uncharacterized HIV-1 recombinant strains. The findings suggested that each of these HIV-1 recombinant putative transmission clusters could be further characterized into novel CRFs.

Then, separate phylogenetic analyses were performed on the 16 near-full-length HIV-1 genome (790–8795 in the HXB2 genome) and two partial genome (790–5250 and 2253–8795 in the HXB2 genome) nucleotide sequences obtained. The generated maximum likelihood trees explicitly show that none of the HIV-1 recombinant sequences clustered with the reference sequences of HIV-1 group M subtypes or previously established CRFs. Instead, in each maximum likelihood tree, the HIV-1 recombinant query sequences exclusively formed distinct clusters with a high bootstrap support of 100%, showing their uniqueness, which suggests that these sequences could belong to a novel HIV-1 CRF lineage.

### 3.3. Description of the Four Novel CRFs

#### 3.3.1. CRF129_56G

**Figure 2:**
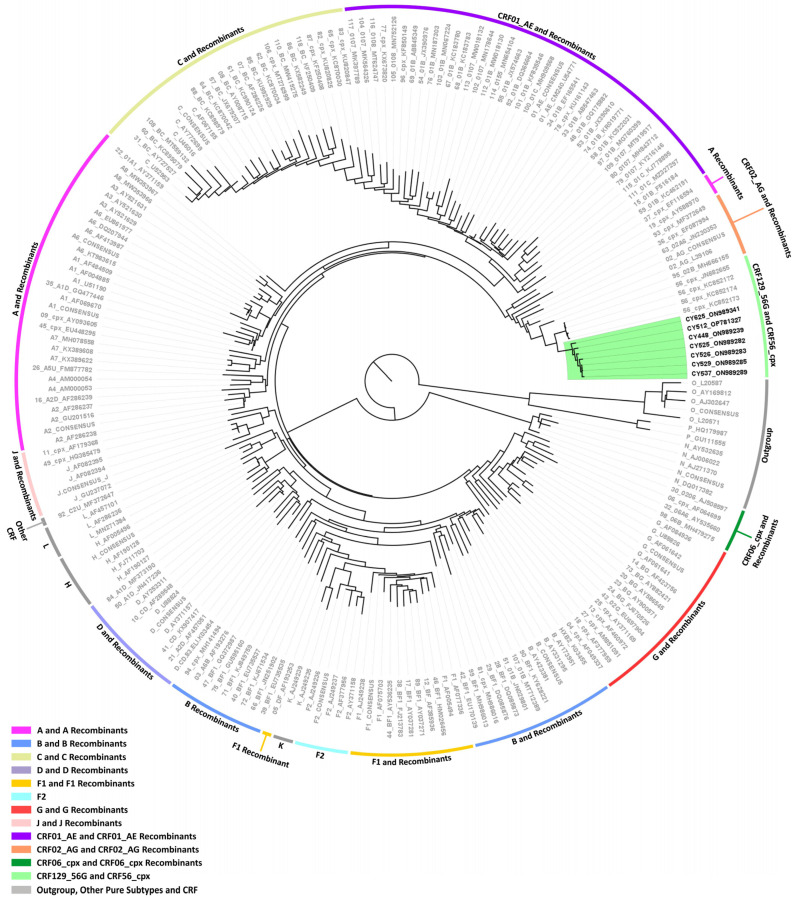
Maximum likelihood phylogenetic tree analyses of the near-full-length HIV-1 genome (790–8795 in the HXB2 genome) nucleotide sequences of the seven HIV-1 recombinants subtyped as “Rec. of B, A1, G” based on the HIV-1 *pol* region (2253–5250 in the HXB2 genome). The seven HIV-1 recombinant sequences were isolated from whole blood samples of seven respective individuals infected with HIV-1 collected in Cyprus, as part of a prospective molecular epidemiology study (C. Topcu et al., manuscript in preparation for publication) [21] conducted from 9 March 2017 to 14 October 2021. Of the seven individuals infected with HIV-1, six were newly diagnosed and one was chronically infected with HIV-1, while all were antiretroviral-naïve. The phylogenetic analyses were executed against a reference dataset of all known HIV-1 group M subtypes (A, B, C, D, F, G, H, J, K, and L) and circulating recombinant forms (CRFs) (RIP Alignment 2020) downloaded from the Los Alamos HIV Sequence Database (http://www.hiv.lanl.gov (accessed on 13 November 2023)) using MEGA X software. The HIV-1 clades are color coded at the periphery of the phylogenetic tree in accordance with the HIV-1 genotypic subtypes of the reference sequences, which are also denoted next to each HIV-1 clade. The color coding is also described on the bottom left of the figure. Reference sequences, labeled in grey, are titled to present the HIV-1 genotypic subtype, followed by the GenBank accession number. The seven HIV-1 recombinant query sequences from Cyprus, labeled in black, are titled to present their unique laboratory identification number, in which the prefix CY is followed by a number denoting the laboratory code, along with their GenBank accession number. The branches highlighted with the color green display the HIV-1 recombinant putative transmission cluster, consisting of the samples CY448, CY512, CY525, CY526, CY529, CY537, and CY625. All seven of these samples making up the HIV-1 recombinant putative transmission cluster were used to characterize the novel CRF129_56G strain.

**Figure 3:**
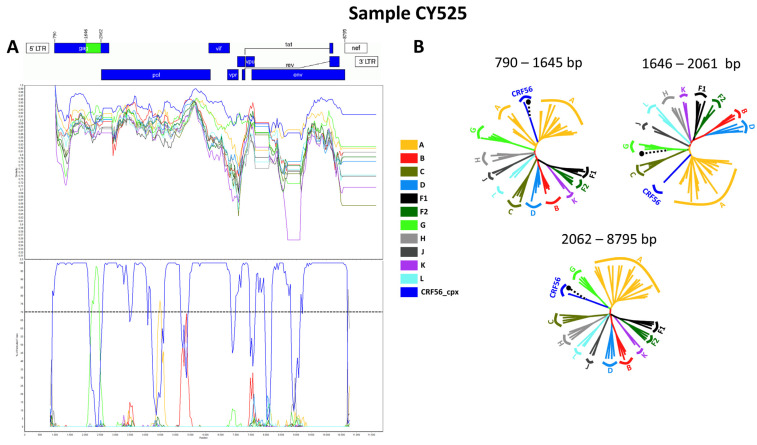
Recombination analyses of the near-full-length HIV-1 genome (790–8795 in the HXB2 genome) nucleotide sequence derived from sample CY525 used as a representative sample to illustrate the unique intersubtype mosaic structure of the CRF129_56G strain. The recombination and phylogenetic analyses were conducted against a reference dataset of HIV-1 group M subtypes (A, B, C, D, F, G, H, J, K, and L), and CRF56_cpx downloaded from the Los Alamos HIV Sequence Database (http://www.hiv.lanl.gov (accessed on 13 November 2023)). The reference dataset used for the recombination analyses was enriched through Basic Local Alignment Search Tool (BLAST) analyses (https://www.hiv.lanl.gov/content/sequence/BASIC_BLAST/basic_blast.html (accessed on 13 November 2023)) with three of the top BLAST hits for CRF56_cpx strain. (**A**) The upper left illustration demonstrates the genomic map of sample CY525, as created using the Recombinant HIV-1 Drawing Tool (https://www.hiv.lanl.gov/content/sequence/DRAW_CRF/recom_mapper.html (accessed on 13 November 2023)). The numbers above the illustration denote the intersubtype recombination breakpoints with respect to the HXB2 genome. The near-full-length HIV-1 genome was divided into three fragments based on the two recombination breakpoints, presenting its unique intersubtype mosaic structure. The subtype origin of each fragment is color coded in agreement with all instructive recombination analyses, and the color coding is described in the middle of the figure. The middle left illustration presents the similarity plot analysis performed using SimPlot v3.5.1 software, where the y-axis shows the percent similarity of the query sequence to the reference dataset. The intermittent horizontal line on the similarity plot depicts the 90% similarity. The lower left illustration presents the bootscan analysis performed using SimPlot v3.5.1 software, in which the y-axis shows the bootstrap support value. The intermittent horizontal line on the bootscan depicts the threshold of 70% bootstrap support value that was decided to be definitive for subtype origin of each fragment. The x-axes in both illustrations signify the loci with respect to the HXB2 genome. Decimal signs in the y-axis are represented with commas, while four and five-digit numbers in the x-axis are displayed with periods. (**B**) The illustration on the right side of the figure displays the subregion confirmatory neighbor-joining tree analyses. MEGA X software was used to construct neighbor-joining trees for each of the fragments as defined by the prior similarity plot and bootscan analyses [25]. Threshold of 70% bootstrap support value was decided to be definitive for subtype origin of each fragment. The loci of the beginning and end of each fragment in accordance with the HXB2 numbering are indicated above each respective phylogenetic tree. The branch displayed by a black intermittent line ending in a black dot demonstrates the query sequence. The color coding of the phylogenetic trees agrees with rest of the recombination analyses within the figure. The GenBank accession numbers of the reference sequences used for these analyses are as follows: A1-AF004885, A1-AF069670, A1-U51190, A1-AF484509; A2-AF286238, A2-GU201516, A2-AF286237; A3-AY521629, A3-AY521630, A3-AY521631; A4-AM000053, A4-AM000054; A6-KT983615, A6-AF413987, A6-DQ207944, A6-EU861977; A7-KX389622, A7-KX389608, A7-MH078558; A8-MW353966, A8-MW353967; B-K03455, B-AY173951, B-AY423381, B-AY331295; C-U52953, C-U46016, C-AF067155, C-AY772699; D-K03454, D-U88824, D-AY253311, D-AY371157; F1-AF077336, F1-AF005494, F1-AF075703, F1-AJ249238; F2-AY371158, F2-AF377956, F2-AJ249237, F2-AJ249236; G-AF084936, G-U88826, G-AF061642, G-AF061641; H-FJ711703, H-AF190127, H-AF190128, H-AF005496; J-GU237072, J-AF082395, J-AF082394; K-AJ249235, K-AJ249239; L-MN271384, L-AF286236, L-AF457101; CRF56_cpx-JN882655, CRF56_cpx-KC852172, CRF56_cpx-KC852173, and CRF56_cpx-KC852174.

Seven out of eight near-full-length HIV-1 genome (790–8795 in the HXB2 genome) nucleotide sequences derived from samples CY448, CY512, CY525, CY526, CY529, CY537, and CY625 were submitted to bootscan and similarity plot analyses. These analyses provided verification of the existence of a new series of recombination events that had not been documented before among the established CRFs. The uniqueness of the HIV-1 recombinants was also evident in their exclusive clustering within the generated maximum likelihood tree shown in Figure 2. As a result, each of the seven HIV-1 recombinant query sequences was assembled of two CRF56_cpx fragments and one subtype G fragment. Figure 3A demonstrates the mosaic genomic map along with the bootscan and similarity plot for sample CY525, which serves as the representative sample for this particular recombinant putative transmission cluster. The schematics showing the findings for the remaining six samples are included in Figure S1 of the Supplementary Materials. The three fragments were divided by two putative intersubtype recombination breakpoints. The two potential intersubtype recombination breakpoints (beginning from the 5′ end) were identified at nucleotide positions 1758 ± 112 and 2037 ± 25, as indicated in the HXB2 genome (Figure 3A).

The confirmatory neighbor-joining tree analyses of subregions additionally validated the two intersubtype recombination breakpoints and confirmed the subtype origin of each of the three fragments that were divided based on the defined breakpoints (Figure 3B). The schematics showing the findings for the remaining six samples are included in Figure S1 of the Supplementary Materials. The phylogenetic investigations showed that the initial fragment (790–1645 or 790–1870 in the HXB2 genome) was closely associated with CRF56_cpx, with robust 100% bootstrap support. The second fragment (1646–2061 or 1871–2011 in the HXB2 genome) exhibited clustering with subtype G, supported by bootstrap values ranging from 72% to 98%. Last, the third fragment (2062–8795 or 2012–8795 in the HXB2 genome) also showed a close association with CRF56_cpx, again with a strong bootstrap support of 100%. Consequently, the mosaic configuration of these seven HIV-1 recombinants was delineated and officially designated CRF129_56G by the Los Alamos HIV Sequence Database, following the established HIV nomenclature guidelines (https://www.hiv.lanl.gov/content/sequence/HelpDocs/subtypes-more.html (accessed on 13 November 2023)).

#### 3.3.2. CRF130_A1B

**Figure 4:**
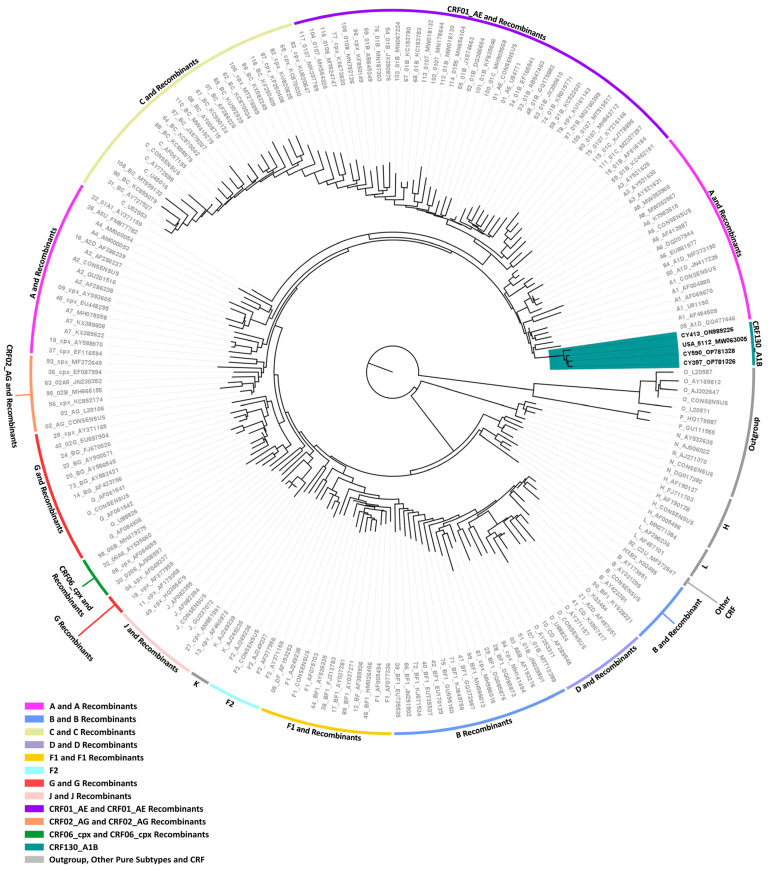
Maximum likelihood phylogenetic tree analyses of two near-full-length HIV-1 genome (790–8795 in the HXB2 genome) and one partial HIV-1 genome (790–5250 in the HXB2 genome) nucleotide sequences of the three HIV-1 recombinants, subtyped either as “Rec. of B, A1” or “Rec. B, A1, F1” based on the HIV-1 *pol* region (2253–5250 in the HXB2 genome), as well as the isolate 5112 (GenBank accession number: MW063005), which was sampled in the United States of America (USA) and was identified through Basic Local Alignment Search Tool (BLAST) analyses (https://www.hiv.lanl.gov/content/sequence/BASIC_BLAST/basic_blast.html (accessed on 13 November 2023)). The three HIV-1 recombinant sequences were isolated from whole blood samples of three respective individuals infected with HIV-1 collected in Cyprus. One of three samples was collected as part of a prospective molecular epidemiology study (C. Topcu et al., manuscript in preparation for publication) [21] conducted from 9 March 2017 to 14 October 2021, and the remaining two were collected as part of the routine HIV-1 genotypic drug resistance analyses performed by our laboratory within the same time period. Of the three individuals, all were chronically infected with HIV-1. One of them that derived from the prospective molecular epidemiology study was antiretroviral-naïve, while the other two individuals were not. The phylogenetic analyses were executed against a reference dataset of all known HIV-1 group M subtypes (A, B, C, D, F, G, H, J, K, and L) and circulating recombinant forms (CRFs) (RIP Alignment 2020) downloaded from the Los Alamos HIV Sequence Database (http://www.hiv.lanl.gov (accessed on 13 November 2023)) using MEGA X software. The HIV-1 clades are color coded at the periphery of the phylogenetic tree in accordance with the HIV-1 genotypic subtypes of the reference sequences, which are also denoted next to each HIV-1 clade. The color coding is also described on the bottom left of the figure. Reference sequences, labeled in grey, are titled to present the HIV-1 genotypic subtype, followed by the GenBank accession number. The three HIV-1 recombinant query sequences from Cyprus, labeled in black, are titled to present their unique laboratory identification number, in which the prefix CY is followed by a number denoting the laboratory code, along with their GenBank accession number. The branches highlighted with the color teal display the HIV-1 recombinant putative transmission cluster, consisting of the samples CY397, CY413, and CY590, as well as the isolate 5112. All four of these samples making up the HIV-1 recombinant putative transmission cluster were used to characterize the novel CRF130_A1B strain.

Two near-full-length HIV-1 genome (790–8795 in the HXB2 genome) nucleotide sequences derived from samples CY397 and CY413 and one HIV-1 partial genome (790–5250 in the HXB2 genome) nucleotide sequence derived from sample CY590 were submitted to bootscan and similarity plot analyses. The analyses have verified the existence of a previously unrecorded series of recombination events within the established CRFs. The uniqueness of the HIV-1 recombinants was also evident in their exclusive clustering within the generated maximum likelihood tree shown in Figure 4. In addition, BLAST analyses for the investigation of other sequences that might have connections with the HIV-1 recombinant query sequences revealed one sequence that exhibits a similar intersubtype mosaicism pattern. The detailed intersubtype recombination analyses showed that isolate 5112 (GenBank accession number: MW063005), which was sampled in the United States of America (USA) between 2018 and 2019, demonstrates the same mosaic structure as the three HIV-1 recombinant query sequences from Cyprus [35].

**Figure 5:**
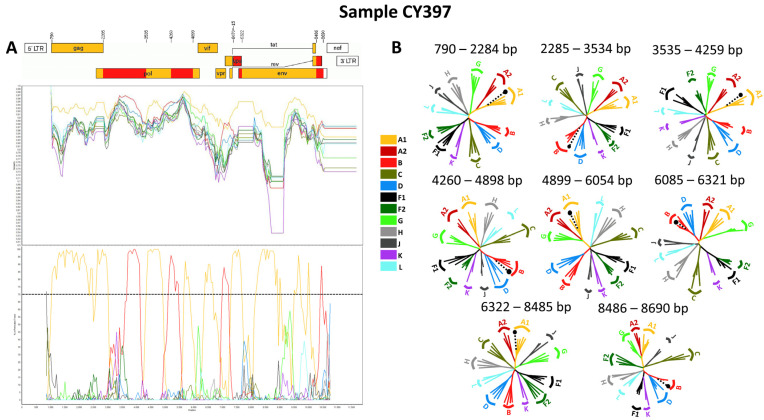
Recombination analyses of the near-full-length HIV-1 genome (790–8795 in the HXB2 genome) nucleotide sequence derived from sample CY397 used as a representative sample to illustrate the unique intersubtype mosaic structure of the CRF130_A1B strain. The recombination and phylogenetic analyses were conducted against a reference dataset of HIV-1 group M subtypes (A1, A2, B, C, D, F, G, H, J, K, and L) downloaded from the Los Alamos HIV Sequence Database (http://www.hiv.lanl.gov (accessed on 13 November 2023)). The reference dataset used for the recombination analyses was enriched through Basic Local Alignment Search Tool (BLAST) analyses (https://www.hiv.lanl.gov/content/sequence/BASIC_BLAST/basic_blast.html (accessed on 13 November 2023)) with 18 of the top BLAST hits for subtype B. (**A**) The upper left illustration demonstrates the genomic map of sample CY397, as created using the Recombinant HIV-1 Drawing Tool (https://www.hiv.lanl.gov/content/sequence/DRAW_CRF/recom_mapper.html (accessed on 13 November 2023)). The numbers above the illustration denote the intersubtype recombination breakpoints with respect to the HXB2 genome. The near-full-length HIV-1 genome was divided into eight fragments based on the seven recombination breakpoints, presenting its unique intersubtype mosaic structure. The subtype origin of each fragment is color coded in agreement with all instructive recombination analyses, and the color coding is described in the middle of the figure. The middle left illustration presents the similarity plot analysis performed using SimPlot v3.5.1 software, where the y-axis shows the percent similarity of the query sequence to the reference dataset. The intermittent horizontal line on the similarity plot depicts the 90% similarity. The lower left illustration presents the bootscan analysis performed using SimPlot v3.5.1 software, in which the y-axis shows the bootstrap support value. The intermittent horizontal line on the bootscan depicts the threshold of 70% bootstrap support value that was decided to be definitive for subtype origin of each fragment. The x-axes in both illustrations signify the loci with respect to the HXB2 genome. Decimal signs in the y-axis are represented with commas, while four and five-digit numbers in the x-axis are displayed with periods. (**B**) The illustration on the right side of the figure displays the subregion confirmatory neighbor-joining tree analyses. MEGA X software was used to construct neighbor-joining trees for each of the fragments as defined by the prior similarity plot and bootscan analyses [25]. Threshold of 70% bootstrap support value was decided to be definitive for subtype origin of each fragment. The loci of the beginning and end of each fragment in accordance with the HXB2 numbering are indicated above each respective phylogenetic tree. The branch displayed by a black intermittent line ending in a black dot demonstrates the query sequence. The color coding of the phylogenetic trees agrees with rest of the recombination analyses within the figure. The GenBank accession numbers of the reference sequences used for these analyses are as follows: A1-AF004885, A1-AF069670, A1-U51190, A1-AF484509; A2-AF286238, A2-GU201516, A2-AF286237; B-K03455, B-AY173951, B-AY423381, B-AY331295, B-DQ076814, B-JX147100, B-DQ076812, B-GU330270, B-GU330253, B-KM355143, B-KT339988, B-KM355099, B-KM355145, B-KX129256, B-KM355092, B-JF683781, B-KM355154, B-KM355116, B-KM355146, B-KM355144, B-KM355149, B-JF683781; C-U52953, C-U46016, C-AF067155, C-AY772699; D-K03454, D-U88824, D-AY253311, D-AY371157; F1-AF077336, F1-AF005494, F1-AF075703, F1-AJ249238; F2-AY371158, F2-AF377956, F2-AJ249237, F2-AJ249236; G-AF084936, G-U88826, G-AF061642, G-AF061641; H-FJ711703, H-AF190127, H-AF190128, H-AF005496; J-GU237072, J-AF082395, J-AF082394; K-AJ249235, K-AJ249239; L-MN271384, L-AF286236, and L-AF457101.

Consequently, each of the near-full-length HIV-1 recombinant query sequences consisted of four fragments of subtype A1 and four fragments of subtype B. Meanwhile, the partial HIV-1 recombinant query sequence was determined to agree with these findings in the regions that were available. Figure 5A displays the mosaic genomic map accompanied by the bootscan and similarity plot for sample CY397, which represents this particular recombinant putative transmission cluster. Additional schematics depicting the findings for the other two Cypriot and one USA sample can be found in Figure S1 of the Supplementary Materials. The eight fragments were divided by seven putative identical intersubtype recombination breakpoints. The seven potential intersubtype recombination breakpoints (beginning from the 5′ end) were identified at nucleotide positions 2285, 3535, 4260, 4899, 6070 ± 15, 6322, and 8486, as indicated in the HXB2 genome (Figure 5A).

The confirmatory neighbor-joining tree analyses of subregions not only further validated the presence of seven intersubtype recombination breakpoints but also confirmed the subtype origin of each of the eight fragments, which were divided based on these defined breakpoints, as shown in Figure 5B. Additional schematics depicting the findings for the other two Cypriot and one USA sample can be found in Figure S1 of the Supplementary Materials. The phylogenetic analyses showed that the initial fragment (790–2284 in the HXB2 genome) was closely associated with subtype A1, with strong 100% bootstrap support. The second fragment (2285–3534 in the HXB2 genome) demonstrated clustering with subtype B, supported by bootstrap values ranging from 93% to 98%. The third fragment (3535–4259 in the HXB2 genome) also showed a close association with subtype A1, again with a robust bootstrap support of 100%. The fourth fragment (4260–4898 in the HXB2 genome) displayed clustering with subtype B, with bootstrap support values varying between 86 and 93%. The fifth fragment (4899–6054 or 4899–6084 in the HXB2 genome) clustered with subtype A1 with bootstrap support values alternating between 97% and 98%. The sixth fragment (6055–6321 or 6085–6321 in the HXB2 genome) was discovered to be associated with subtype B with bootstrap support values differing between 83% and 86%. The seventh fragment (6322–8485 in the HXB2 genome) was strongly associated with subtype A1 with powerful 100% bootstrap support. Finally, the eighth fragment (8486–8690 in the HXB2 genome) again presented clustering with subtype B, supported by bootstrap values ranging from 83% to 93%. As a result, the mosaic configuration of these four HIV-1 recombinants was depicted and officially designated CRF130_A1B by the Los Alamos HIV Sequence Database, following the established HIV nomenclature guidelines (https://www.hiv.lanl.gov/content/sequence/HelpDocs/subtypes-more.html (accessed on 13 November 2023)).

#### 3.3.3. CRF131_A1B

**Figure 6:**
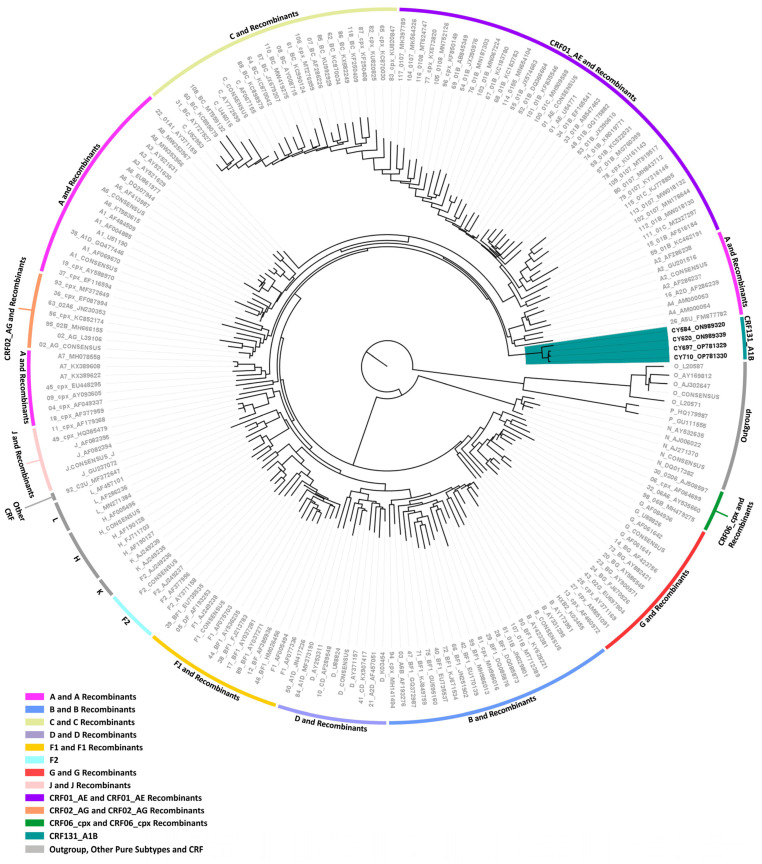
Maximum likelihood phylogenetic tree analyses of the near-full-length HIV-1 genome (790–8795 in the HXB2 genome) nucleotide sequences of the four HIV-1 recombinants subtyped either as “Rec. of B, A1” or “Rec. B, A1, D” based on the HIV-1 *pol* region (2253–5250 in the HXB2 genome). The four HIV-1 recombinant sequences were isolated from whole blood samples of four respective individuals infected with HIV-1, collected in Cyprus as part of a prospective molecular epidemiology study (C. Topcu et al., manuscript in preparation for publication) [21] conducted from 9 March 2017 to 14 October 2021. Of the four individuals infected with HIV-1, all were newly diagnosed and antiretroviral-naïve. The phylogenetic analyses were executed against a reference dataset of all known HIV-1 group M subtypes (A, B, C, D, F, G, H, J, K, and L) and circulating recombinant forms (CRFs) (RIP Alignment 2020) downloaded from the Los Alamos HIV Sequence Database (http://www.hiv.lanl.gov (accessed on 13 November 2023)) using MEGA X software. The HIV-1 clades are color coded at the periphery of the phylogenetic tree in accordance with the HIV-1 genotypic subtypes of the reference sequences, which are also denoted next to each HIV-1 clade. The color coding is also described on the bottom left of the figure. Reference sequences, labeled in grey, are titled to present the HIV-1 genotypic subtype, followed by the GenBank accession number. The four HIV-1 recombinant query sequences from Cyprus, labeled in black, are titled to present their unique laboratory identification number, in which the prefix CY is followed by a number denoting the laboratory code, along with their GenBank accession number. The branches highlighted with the color teal display the HIV-1 recombinant putative transmission cluster, consisting of the samples CY584, CY620, CY697, and CY710. All four of these samples making up the HIV-1 recombinant putative transmission cluster were used to characterize the novel CRF131_A1B strain.

**Figure 7:**
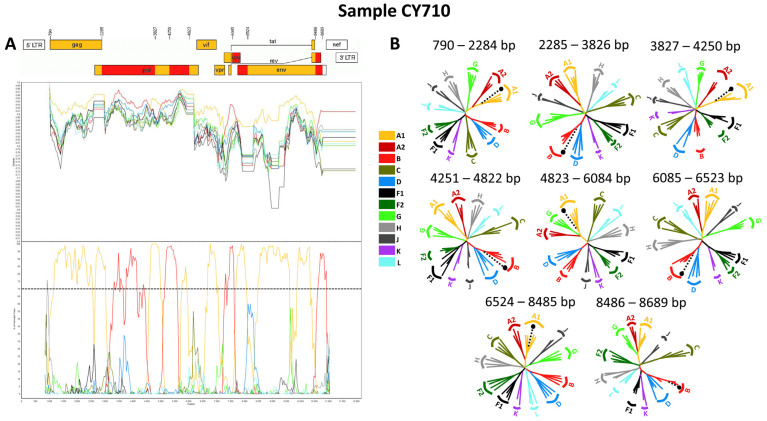
Recombination analyses of the near-full-length HIV-1 genome (790–8795 in the HXB2 genome) nucleotide sequence derived from sample CY710 used as a representative sample to illustrate the unique intersubtype mosaic structure of the CRF131_A1B strain. The recombination and phylogenetic analyses were conducted against a reference dataset of HIV-1 group M subtypes (A1, A2, B, C, D, F, G, H, J, K, and L) downloaded from the Los Alamos HIV Sequence Database (http://www.hiv.lanl.gov (accessed on 13 November 2023)). The reference dataset used for the recombination analyses was enriched through Basic Local Alignment Search Tool (BLAST) analyses (https://www.hiv.lanl.gov/content/sequence/BASIC_BLAST/basic_blast.html (accessed on 13 November 2023)) with 18 of the top BLAST hits for subtype B. (**A**) The upper left illustration demonstrates the genomic map of sample CY710, as created using the Recombinant HIV-1 Drawing Tool (https://www.hiv.lanl.gov/content/sequence/DRAW_CRF/recom_mapper.html (accessed on 13 November 2023)). The numbers above the illustration denote the intersubtype recombination breakpoints with respect to the HXB2 genome. The near-full-length HIV-1 genome was divided into eight fragments based on the seven recombination breakpoints, presenting its unique intersubtype mosaic structure. The subtype origin of each fragment is color coded in agreement with all instructive recombination analyses, and the color coding is described in the middle of the figure. The middle left illustration presents the similarity plot analysis performed using SimPlot v3.5.1 software, where the y-axis shows the percent similarity of the query sequence to the reference dataset. The intermittent horizontal line on the similarity plot depicts the 90% similarity. The lower left illustration presents the bootscan analysis performed using SimPlot v3.5.1 software, in which the y-axis shows the bootstrap support value. The intermittent horizontal line on the bootscan depicts the threshold of 70% bootstrap support value that was decided to be definitive for subtype origin of each fragment. The x-axes in both illustrations signify the loci with respect to the HXB2 genome. Decimal signs in the y-axis are represented with commas, while four and five-digit numbers in the x-axis are displayed with periods. (**B**) The illustration on the right side of the figure displays the subregion confirmatory neighbor-joining tree analyses. MEGA X software was used to construct neighbor-joining trees for each of the fragments as defined by the prior similarity plot and bootscan analyses [25]. Threshold of 70% bootstrap support value was decided to be definitive for subtype origin of each fragment. The loci of the beginning and end of each fragment in accordance with the HXB2 numbering are indicated above each respective phylogenetic tree. The branch displayed by a black intermittent line ending in a black dot demonstrates the query sequence. The color coding of the phylogenetic trees agrees with rest of the recombination analyses within the figure. The GenBank accession numbers of the reference sequences used for these analyses are as follows: A1-AF004885, A1-AF069670, A1-U51190, A1-AF484509; A2-AF286238, A2-GU201516, A2-AF286237; B-K03455, B-AY173951, B-AY423381, B-AY331295, B-DQ076814, B-JX147100, B-DQ076812, B-GU330270, B-GU330253, B-KM355143, B-KT339988, B-KM355099, B-KM355145, B-KX129256, B-KM355092, B-JF683781, B-KM355154, B-KM355116, B-KM355146, B-KM355144, B-KM355149, B-JF683781; C-U52953, C-U46016, C-AF067155, C-AY772699; D-K03454, D-U88824, D-AY253311, D-AY371157; F1-AF077336, F1-AF005494, F1-AF075703, F1-AJ249238; F2-AY371158, F2-AF377956, F2-AJ249237, F2-AJ249236; G-AF084936, G-U88826, G-AF061642, G-AF061641; H-FJ711703, H-AF190127, H-AF190128, H-AF005496; J-GU237072, J-AF082395, J-AF082394; K-AJ249235, K-AJ249239; L-MN271384, L-AF286236, and L-AF457101.

Four near-full-length HIV-1 genome (790–8795 in the HXB2 genome) nucleotide sequences derived from samples CY584, CY620, CY697, and CY710 underwent bootscan and similarity plot analyses. The analyses verified the existence of a previously undocumented series of recombination events among the established CRFs. The uniqueness of the HIV-1 recombinants was also evident in their exclusive clustering within the generated maximum likelihood tree shown in Figure 6. As such, akin to the CRF130_A1B strain, each of the four HIV-1 recombinant query sequences was composed of four subtype A1 fragments and four subtype B fragments. Figure 7A illustrates the mosaic genomic map along with the bootscan and similarity plot for sample CY710, which was employed to represent this recombinant putative transmission cluster. The schematics displaying the findings for the remaining three samples are available in Figure S1 of the Supplementary Materials. The eight fragments were divided by seven putative identical intersubtype recombination breakpoints. The seven potential intersubtype recombination breakpoints (beginning from the 5′ end) were identified at nucleotide positions 2285, 3827, 4251, 4823, 6085, 6524, and 8486, as indicated in the HXB2 genome (Figure 7A).

The confirmatory neighbor-joining tree analyses of subregions provided further validation of the seven identified intersubtype recombination breakpoints and confirmed the subtype origin of each of the eight fragments that were divided based on the defined breakpoints (Figure 7B). The schematics displaying the findings for the remaining three samples are available in Figure S1 of the Supplementary Materials. The phylogenetic evaluations revealed that the initial fragment (790–2284 in the HXB2 genome) was closely linked to subtype A1, with strong 100% bootstrap support. The second fragment (2285–3826 in the HXB2 genome) formed a cluster with subtype B, with bootstrap values varying between 96% and 100%. Similarly, the third fragment (3827–4250 in the HXB2 genome) exhibited a close association with subtype A1, again supported by a robust bootstrap value of 100%. The fourth fragment (4251–4822 in the HXB2 genome) showed clustering with subtype B, with bootstrap values ranging from 86% to 96%. The fifth fragment (4823–6084 in the HXB2 genome) clustered with subtype A1, with bootstrap support values differing between 96% and 100%. The sixth fragment (6085–6523 in the HXB2 genome) was found to be linked with subtype B, with bootstrap support values varying between 94% and 98%. The seventh fragment (6524–8485 in the HXB2 genome) exhibited a strong association with subtype A1, displaying powerful bootstrap support values of 99% to 100%. Last, the eighth fragment (8486–8689 in the HXB2 genome) again clustered with subtype B, supported by bootstrap values ranging from 81% to 92%. Consequently, the mosaic configuration of these four HIV-1 recombinants was delineated and officially designated CRF131_A1B by the Los Alamos HIV Sequence Database, following the established HIV nomenclature guidelines (https://www.hiv.lanl.gov/content/sequence/HelpDocs/subtypes-more.html (accessed on 13 November 2023)).

Specifically, at nucleotide positions 3535–3826 (292 nucleotides) and 6322–6523 (202 nucleotides) based on the HXB2 genome, we observed notable distinctions in the consensus genome of the CRF130_A1B strain, characterized by subtype A1, as opposed to the consensus genome of the CRF131_A1B strain, characterized by subtype B. Notably, only the differences that are more than 200 nucleotides in length are indicated, while there are three other differences in recombination breakpoints between the two strains that are of shorter lengths. These two disparities of considerable lengths encompass a total of 494 nucleotides within their genomes. Notably, the illustration shown in Figure S2 of the Supplementary Materials clearly highlights these differences between the two CRF strains. Additionally, in the maximum likelihood tree depicted in Figure 1, distinct putative transmission clusters are evident for the HIV-1 recombinants of CRF130_A1B and CRF131_A1B strains, highlighting their divergent genetic characteristics. Nevertheless, close clustering of the HIV-1 recombinants of CRF130_A1B and CRF131_A1B suggests a high degree of relatedness between these two novel CRFs. Similarly, our investigation using isolate 5112 (GenBank accession number: MW063005), which was a top BLAST hit with high percent similarity during the BLAST analyses for both CRF strains, revealed intriguing findings [35]. While subsequent recombination analyses unveiled its sharing of the same intersubtype recombination pattern as CRF130_A1B, we employed this sequence as a query to compare it with HIV-1 recombinant sequences from both CRF130_A1B and CRF131_A1B strains. The objective was to assess whether recombination analyses could discern subtle differences between the two strains and accurately categorize isolate 5112. Remarkably, this was successful. The comprehensive results of this analysis are presented in Figure S3 of the Supplementary Materials.

#### 3.3.4. CRF138_cpx

**Figure 8:**
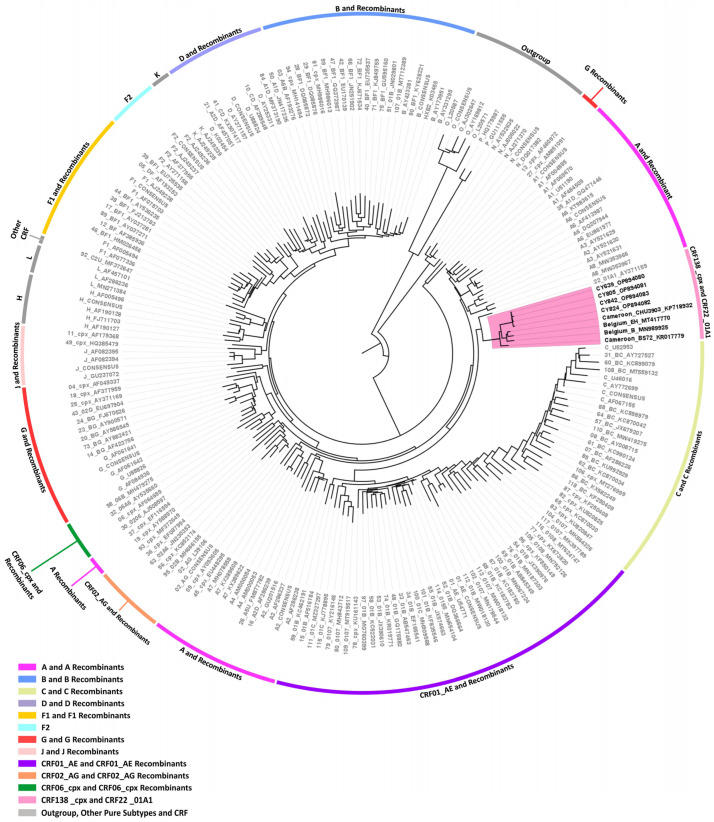
Maximum likelihood phylogenetic tree analyses of three near-full-length HIV-1 genome (790–8795 in the HXB2 genome) and one partial HIV-1 genome (2253–8795 in the HXB2 genome) nucleotide sequences of the four HIV-1 recombinants subtyped as “Rec. of A1, F1” based on the HIV-1 *pol* region (2253–5250 in the HXB2 genome), as well as the isolate B (GenBank accession number: MN989925), isolate EH (GenBank accession number: MT417770), isolate CHU3903 (GenBank accession number: KP718932), and isolate BS72 (GenBank accession number: KR017779) that were identified through Basic Local Alignment Search Tool (BLAST) analyses (https://www.hiv.lanl.gov/content/sequence/BASIC_BLAST/basic_blast.html (accessed on 13 November 2023)). Specifically, the isolates B and EH were sampled in Belgium, while isolates CHU3903 and BS72 were sampled in Cameroon. The four HIV-1 recombinant sequences were isolated from whole blood samples of four respective individuals infected with HIV-1, collected in Cyprus as part of a prospective molecular epidemiology study (C. Topcu et al., manuscript in preparation for publication) [21] conducted from 9 March 2017 to 14 October 2021. Of the four individuals infected with HIV-1, all were newly diagnosed and antiretroviral-naïve. The phylogenetic analyses were executed against a reference dataset of all known HIV-1 group M subtypes (A, B, C, D, F, G, H, J, K, and L) and circulating recombinant forms (CRFs) (RIP Alignment 2020) downloaded from the Los Alamos HIV Sequence Database (http://www.hiv.lanl.gov) using MEGA X software. The HIV-1 clades are color coded at the periphery of the phylogenetic tree in accordance with the HIV-1 genotypic subtypes of the reference sequences, which are also denoted next to each HIV-1 clade. The color coding is also described on the bottom left of the figure. Reference sequences, labeled in grey, are titled to present the HIV-1 genotypic subtype, followed by the GenBank accession number. The four HIV-1 recombinant query sequences from Cyprus, labeled in black, are titled to present their unique laboratory identification number, in which the prefix CY is followed by a number denoting the laboratory code, along with their GenBank accession number. The branches highlighted with the color pink display the HIV-1 recombinant putative transmission cluster, consisting of the samples CY639, CY805, CY824, and CY842, as well as the isolates B, EH, CHU3903, and BS72. Seven of these eight samples making up the HIV-1 recombinant putative transmission cluster were used to characterize the novel CRF138_cpx strain, whereas the remaining sample, isolate BS72, that was identified through the BLAST analyses, was characterized as the unique recombinant from (URF) of CRF138_cpx, “Rec. of 138_cpx, F2, U”. Isolate BS72 also appears within the HIV-1 recombinant putative transmission cluster highlighted in pink, presenting its high genetic similarity to the CRF138_cpx strain.

Three near-full-length HIV-1 genome (790–8795 in the HXB2 genome) nucleotide sequences derived from samples CY639, CY805, and CY824 and one HIV-1 partial genome (2253–8795 in the HXB2 genome) nucleotide sequence derived from sample CY842 were submitted to bootscan and similarity plot analyses. The analyses provided validation of the existence of a new series of recombination events that had not been recorded before among the established CRFs. The uniqueness of the HIV-1 recombinants was also evident in their exclusive clustering within the generated maximum likelihood tree shown in Figure 8. Additionally, when conducting BLAST analyses to explore sequences potentially related to the HIV-1 recombinant query sequences, we identified four sequences that exhibit similar intersubtype mosaicism patterns. Detailed analyses of intersubtype recombination revealed that three of the four sequences, isolate B (GenBank accession number: MN989925) and isolate EH (GenBank accession number: MT417770), both sampled in Belgium in 2019, as well as isolate CHU3903 (GenBank accession number: KP718932), sampled in Cameroon in 2011, share the same mosaic structure as the four HIV-1 recombinant query sequences from Cyprus [36,37]. 

**Figure 9:**
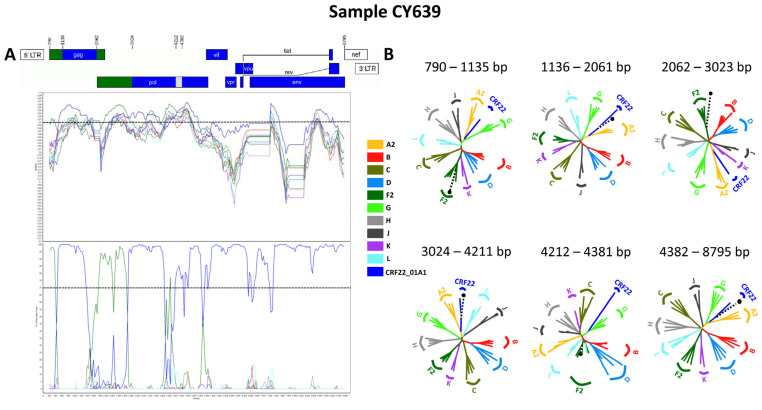
Recombination analyses of the near-full-length HIV-1 genome (790–8795 in the HXB2 genome) nucleotide sequence derived from sample CY639 used as a representative sample to illustrate the unique intersubtype mosaic structure of the CRF138_cpx strain. The recombination and phylogenetic analyses were conducted against a reference dataset of HIV-1 group M subtypes (A2, B, C, D, F2, G, H, J, K, and L) and CRF22_01A1 downloaded from the Los Alamos HIV Sequence Database (http://www.hiv.lanl.gov (accessed on 13 November 2023)), which was enriched through Basic Local Alignment Search Tool (BLAST) analyses (https://www.hiv.lanl.gov/content/sequence/BASIC_BLAST/basic_blast.html (accessed on 13 November 2023)), with six of the top BLAST hits for sub-subtype F2 and CRF22_01A1. (**A**) The upper left illustration demonstrates the genomic map of sample CY710, as created using the Recombinant HIV-1 Drawing Tool (https://www.hiv.lanl.gov/content/sequence/DRAW_CRF/recom_mapper.html (accessed on 13 November 2023)). The numbers above the illustration denote the intersubtype recombination breakpoints with respect to the HXB2 genome. The near-full-length HIV-1 genome was divided into six fragments based on the five recombination breakpoints, presenting its unique intersubtype mosaic structure. The subtype origin of each fragment is color coded in agreement with all instructive recombination analyses, and the color coding is described in the middle of the figure. The middle left illustration presents the similarity plot analysis performed using SimPlot v3.5.1 software, where the y-axis shows the percent similarity of the query sequence to the reference dataset. The intermittent horizontal line on the similarity plot depicts the 90% similarity. The lower left illustration presents the bootscan analysis performed using SimPlot v3.5.1 software, in which the y-axis shows the bootstrap support value. The intermittent horizontal line on the bootscan depicts the threshold of 70% bootstrap support value that was decided to be definitive for subtype origin of each fragment. The x-axes in both illustrations signify the loci with respect to the HXB2 genome. Decimal signs in the y-axis are represented with commas, while four-digit numbers in the x-axis are displayed with periods. (**B**) The illustration on the right side of the figure displays the subregion confirmatory neighbor-joining tree analyses. MEGA X software was used to construct neighbor-joining trees for each of the fragments as defined by the prior similarity plot and bootscan analyses [25]. Threshold of 70% bootstrap support value was decided to be definitive for subtype origin of each fragment. The loci of the beginning and end of each fragment in accordance with the HXB2 numbering are indicated above each respective phylogenetic tree. The branch displayed by a black intermittent line ending in a black dot demonstrates the query sequence. The color coding of the phylogenetic trees agrees with rest of the recombination analyses within the figure. The GenBank accession numbers of the reference sequences used for the bootscan and similarity plot analyses are as follows: B-K03455, B-AY173951, B-AY423381, B-AY331295; C-U52953, C-U46016, C-AF067155, C-AY772699; D-K03454, D-U88824, D-AY253311, D-AY371157; F2-AY371158, F2-AJ249237, F2-AJ249236, F2-KR822828; H-FJ711703, H-AF190127, H-AF190128, H-AF005496; J-GU237072, J-AF082395, J-AF082394; K-AJ249239; L-AF457101; CRF22_01A1-KF716460, CRF22_01A1-KX228816, CRF22_01A1-KF716462, CRF22_01A1-KP109499, and CRF22_01A1-JN864050. The GenBank accession numbers of the reference sequences used for the confirmatory phylogenetic tree analyses of subregions are as follows: B-K03455, B-AY173951, B-AY423381, B-AY331295; C-U52953, C-U46016, C-AF067155, C-AY772699; D-K03454, D-U88824, D-AY253311, D-AY371157; F2-AY371158, F2-AF377956, F2-AJ249237, F2-AJ249236; G-AF084936, G-U88826, G-AF061642, G-AF061641; H-FJ711703, H-AF190127, H-AF190128, H-AF005496; J-GU237072, J-AF082395, J-AF082394; K-AJ249235, K-AJ249239; L-MN271384, L-AF286236, L-AF457101; and CRF22_01A1-AY371159.

Accordingly, each of the near-full-length HIV-1 recombinant query sequences were assembled of three fragments of CRF22_01A1, two fragments of subtype F2, and a fragment of unknown subtype origin. Meanwhile, the partial HIV-1 recombinant query sequence was determined to agree with these findings in the regions that were available. Figure 9A exhibits the mosaic genomic map accompanied by the bootscan and similarity plot for sample CY639, which was chosen to represent this particular recombinant putative transmission cluster. The schematics presenting the findings for the other three Cypriot, two Belgian, and one Cameroonian sample can be observed in Figure S1 of the Supplementary Materials. The six fragments were divided by five putative intersubtype recombination breakpoints. The five potential intersubtype recombination breakpoints (beginning from the 5′ end) were identified at nucleotide positions 1138 ± 6, 2062, 3024, 4261 ± 49, and 4382 ± 24, as indicated in the HXB2 genome (Figure 9A).

The confirmatory neighbor-joining tree analyses of subregions also confirmed the five intersubtype recombination breakpoints and validated the previously identified subtype origin of each of the six fragments that were divided based on the defined breakpoints (Figure 9B). The schematics presenting the findings for the remaining three samples can be observed in Figure S1 of the Supplementary Materials. The phylogenetic examinations revealed that the initial fragment (790–1131, 790–1135 or 790–1143 in the HXB2 genome) clustered with subtype F2, which was supported by bootstrap values ranging from 95% to 99%. The second fragment (1132–2061, 1136–2061 or 1144–2061 in the HXB2 genome) was closely associated with CRF22_01A1, with robust bootstrap support values alternating between 99% and 100%. The third fragment (2062–3023 in the HXB2 genome) also showed a close association with subtype F2 with strong bootstrap support of 100%. The fourth fragment (3024–4211, 3024–4284 or 3024–4309 in the HXB2 genome) was strongly linked to CRF22_01A1, again with powerful 100% bootstrap support. However, the fifth fragment (4212–4357, 4212–4381, 4212–4406, 4285–4406 or 4310–4406 in the HXB2 genome) did not exhibit clustering with any of the pure subtypes or CRFs above the threshold of 70% bootstrap support, which was decided to be definitive for the subtype origin of each fragment. The bootstrap support values ranged between 8% and 58%, with alternating clustering patterns observed between reference sequences for subtype F2 and CRF22_01A1. Finally, the sixth fragment (4358–8795, 4382–8795 or 4407–8795 in the HXB2 genome) was discovered to be associated with CRF22_01A1, with robust bootstrap support of 100%. As a result, the mosaic configuration of these seven HIV-1 recombinants was delineated and officially designated CRF138_cpx by the Los Alamos HIV Sequence Database, in accordance with established HIV nomenclature guidelines (https://www.hiv.lanl.gov/content/sequence/HelpDocs/subtypes-more.html (accessed on 13 November 2023)).

Consequently, the detailed analyses of intersubtype recombination revealed that one of the four sequences identified through BLAST analyses, isolate BS72 (GenBank accession number: KR017779), displayed a slightly distinct mosaic structure [38]. This particular HIV-1 recombinant isolate was made up of four CRF22_01A1 fragments, three subtype F2 fragments, and two fragments with unknown subtype origins. Figure S4A in the Supplementary Materials provides a visual representation of the mosaic genomic map, along with the bootscan and similarity plots for this isolate. These nine fragments were divided by eight potential intersubtype recombination breakpoints. These eight identified intersubtype recombination breakpoints (beginning from the 5′ end) were located at nucleotide positions 1144, 2062, 3024, 3324, 3764, 4212, 4432, and 8358, as indicated in the HXB2 genome (Figure S4A). Isolate BS72 shared the same composition of subtypes and intersubtype recombination breakpoints as CRF138_cpx but had three additional recombination sites. These additional sites were due to the recombination of subtype F2 within the fourth fragment (3324–3763 in the HXB2 genome) and the recombination of a fragment with an unknown subtype origin at the end of the sixth fragment (8358–8795 in the HXB2 genome) within the genome of CRF138_cpx. This indicates that isolate BS72 can be classified as a URF of CRF138_cpx with subtype F2 and a fragment of unknown subtype origin.

The confirmatory neighbor-joining tree analyses confirmed the eight potential intersubtype recombination breakpoints and verified the previously identified subtypes of origin for each of the nine fragments that were divided based on the defined breakpoints (Figure S5B). Accordingly, the phylogenetic investigations revealed that the initial fragment (790–1143 in the HXB2 genome), the second fragment (1144–2061 in the HXB2 genome), and the third fragment (2062–3023 in the HXB2 genome) of the CRF138_cpx URF clustered with subtype F2, CRF22_01A1, and subtype F2, respectively. These clusters displayed the same mosaic structure as CRF138_cpx, with bootstrap support values of 91%, 100%, and 100%, respectively. The fourth fragment (3024–3323 in the HXB2 genome) was closely associated with CRF22_01A1, supported by a strong 99% bootstrap support value. The fifth fragment (3324–3763 in the HXB2 genome) clustered with subtype F2 with 94% bootstrap support, indicating the first difference between CRF138_cpx and its URF. The sixth fragment (3764–4211 in the HXB2 genome) was also closely associated with CRF22_01A1, with bootstrap support of 99%. However, the seventh fragment (4212–4431 in the HXB2 genome), similar to CRF138_cpx, did not exhibit clustering with any known pure subtypes or CRFs above the threshold of a 70% bootstrap support value. It clustered with reference sequences for subtype F2 but with bootstrap support of 20%. The eighth fragment (4432–8357 in the HXB2 genome) was strongly linked to CRF22_01A1, with powerful 100% bootstrap support. Finally, much like the seventh fragment, the ninth fragment (8358–8795 in the HXB2 genome), which represents the second difference between CRF138_cpx and its URF, did not exhibit clustering with any of the pure subtypes or CRFs above the threshold of 70% bootstrap support. It clustered with reference sequences for subtype F2 but with a bootstrap support of 51%. To clearly demonstrate the three additional recombination sites and the two additional fragments within the genome of CRF138_cpx URF as opposed to the genome of CRF138_cpx, further recombination analyses were conducted. Bootscan and similarity plot analyses were duplicated, followed by including the seven HIV-1 recombinant sequences representing the CRF138_cpx strain in the reference dataset, which highlights the two slight differences between the mosaic genomic structures of isolate BS71, the URF of CRF138_cpx, and the CRF138_cpx strain. This illustration is available in Figure S5 of the Supplementary Materials.

## 4. Discussion

In this research, we analyzed the 16 near-full-length and 2 partial HIV-1 genome nucleotide sequences of 18 HIV-1 recombinant viruses from Cyprus. As a result, we identified and characterized four new HIV-1 CRFs and one URF in Cyprus. Specifically, these novel CRFs were named CRF129_56G, CRF130_A1B, CRF131_A1B, and CRF138_cpx by the Los Alamos HIV Sequence Database according to the naming standards of the HIV nomenclature (https://www.hiv.lanl.gov/content/sequence/HelpDocs/subtypes-more.html (accessed on 13 November 2023)). The study participants from whom the HIV-1 recombinant viruses were isolated had no discernible epidemiological connections to one another. Consequently, the specified criteria outlined by the Los Alamos HIV Sequence Database for establishing a new CRF (https://www.hiv.lanl.gov/content/sequence/HIV/CRFs/data/defining_new_CRFs.html (accessed on 13 November 2023)) were satisfied. Moreover, our study introduces a distinct HIV-1 recombinant that emerged from the newly characterized CRF138_cpx and subtype F2. This unique recombinant, labeled “Rec. of 138_cpx, F2, U”, had not been previously documented and was identified in only one HIV-1 recombinant virus, thus earning the classification of a URF, URF of CRF138_cpx. A thorough examination of the clinical, epidemiological, behavioral, and demographic profiles of the study participants has yielded valuable insights into the characteristics of the study participants infected with the four novel CRFs. The results highlight the predominance of these novel CRFs within the male population, particularly among Cypriot MSM, and subsequently among individuals with HBC, a substantial proportion of whom reported HIV-1 transmission within Cyprus.

These findings expand our understanding of the ever-evolving HIV-1 diversity of the island. Cyprus is facing a complex HIV-1 epidemic marked by a polyphyletic infection. This condition involves an increasing diversity of HIV-1 strains, a pattern that has been revealed in prior comprehensive molecular epidemiology studies [12,13,14,15,17,18,19,20]. In the context of this diverse HIV-1 epidemic, CRF129_56G, CRF130_A1B, CRF131_A1B, and CRF138_cpx are the third, fourth, fifth, and sixth recombinant HIV-1 strains, respectively, to be identified among HIV-1 samples collected in Cyprus. This follows the earlier characterization of CRF04_cpx and CRF91_cpx [11,13]. Indeed, these four newly identified CRFs are pioneering in that they mark the first instances among the 140 CRFs documented in the Los Alamos HIV Sequence Database (https://www.hiv.lanl.gov/content/sequence/HIV/CRFs/crfs.comp (accessed on 13 November 2023)) of emergence as a result of recombination events between parental viruses from the specified lineages. In addition, Cyprus has seen the discovery and characterization of seven other URFs within its HIV-1 isolates, and many more URFs are still uncharacterized [15,17,18].

Regarding CRF129_56G, it is worth noting that this particular CRF featured seven HIV-1 recombinant sequences isolated from seven respective individuals from Cyprus. These seven recombinants exhibited a consistent and exclusive intersubtype mosaic structure. This unique structure was characterized by the presence of three distinct fragments separated by two intersubtype recombination breakpoints, which originated from both the CRF56_cpx and G lineages, combining elements from two different lineages into a novel configuration. One out of eight HIV-1 recombinants belonging to this recombinant putative transmission cluster was excluded due to failure in acquiring the near-full-length HIV-1 genome. However, the partial HIV-1 genome sequence (790–5250 in the HXB2 genome) belonging to this recombinant displayed the same intersubtype mosaic structure as the CRF129_56G strain and was confirmed to belong to this strain as well.

In accordance with the clinical, epidemiological, behavioral, and demographic aspects, an interesting pattern emerged within the CRF129_56G putative transmission cluster. It was exclusively composed of male individuals, with the majority being Cypriot MSM. Most of these individuals reported being infected with the virus in Cyprus and were primarily residing in urban regions of Nicosia, one of the two major epicenters of the HIV-1 epidemic in Cyprus, the other being urban regions of Larnaca, according to the preliminary findings of the prospective molecular epidemiology study (C. Topcu et al., manuscript in preparation for publication) [21]. However, it is worth noting that one individual in the CRF129_56G putative transmission cluster resided in Larnaca. This finding indicates the potential for CRF129_56G transmission to reach the second epicenter of the HIV-1 epidemic, Larnaca, soon, which could contribute to the cluster’s further growth. The intriguing facet of these findings lies in the fact that while CRF129_56G has been mostly circulating within the MSM community in Cyprus, the cluster’s composition, which includes two homo/bisexual and one heterosexual individual, has significant epidemiological implications. This hints at the possibility of CRF129_56G spreading beyond the confines of the MSM network into a heterosexual context. However, it is worth considering that cultural factors and the relatively small size of Cyprus may lead study participants to self-identify as heterosexual while not disclosing their actual sexual behaviors.

Despite Cyprus being the most common country of origin among the cases, it is noteworthy that the CRF129_56G putative transmission cluster also encompassed participants from two additional countries: Lebanon and Greece. Additionally, two individuals stated their country of infection as Greece, suggesting the possibility of the recombinant strain being imported from Greece. However, in the BLAST analyses, we could not find any HIV-1 sequences that shared the same recombinant mosaic structure as the newly characterized CRF129_56G strain, which supports the hypothesis that the recombinant strain was generated on the island. Nevertheless, further assessment of spatiotemporal evolutionary dynamics is needed to better understand the precise location and timeframe of the origin of the CRF129_56G strain. Last, it should be highlighted that this putative transmission cluster remains active according to the ongoing assessment of recent onward transmission of HIV-1 within a cluster depth of less than or equal to five years, which was based on the established methodologies described in a prior publication by our laboratory [14]. However, since the most recent entry to this cluster in August 2019, the recombinant putative transmission cluster has not shown growth. As such, currently, there are eight HIV-1 sequences identified as part of the CRF129_56G strain, all sampled in Cyprus.

Concerning CRF130_A1B, this particular CRF was detected in four HIV-1 recombinants. Among these, three of the recombinants, which are discussed in this paper, were isolated in Cyprus, while the fourth was identified through BLAST analyses as originating from the USA. These four recombinants displayed a consistent and unique intersubtype mosaic arrangement. This unique arrangement consisted of eight separate fragments divided by seven intersubtype recombination breakpoints. These fragments were derived from both the A1 and B lineages, merging elements from these two different lineages into a new mosaic structure. 

With respect to the clinical, epidemiological, behavioral, and demographic data, importantly, the CRF130_A1B putative transmission cluster was made up entirely of Cypriot males, who reported being infected with HIV-1 in Cyprus. Two of the individuals stated that they were homo/bisexual, while one belonged to the MSM community. Additionally, the initial HIV-1 positive test dates for the individuals in this recombinant putative transmission cluster were May 2012, June 2012, and October 2013, indicating that the cluster had formed well before its detection and has been in circulation for a significant duration.

Based on the criteria outlined earlier, it is evident that the CRF130_A1B putative transmission cluster remains active as the most recent entry into this cluster was documented in February 2019. Remarkably, since the characterization of the CRF130_A1B strain, two additional HIV-1 sequences exhibiting the same recombinant mosaicism were incorporated into this recombinant putative transmission cluster in August 2022 and November 2022. These sequences were extracted from two individuals, CY980 and CY1009, respectively, both identified as MSM community members. Both individuals indicated Cyprus as their country of infection. Moreover, isolate 5112 (GenBank accession number: MW063005) collected in the USA between 2018 and 2019, which was identified through BLAST analyses, revealed an intersubtype mosaic structure identical to that of the CRF130_A1B strain [35]. This suggests that the CRF130_A1B strain may have been exported from Cyprus to the USA, considering the sampling years. However, a more thorough examination of spatiotemporal evolutionary dynamics is necessary to precisely determine the origin and timeline of the CRF130_A1B strain. Consequently, six HIV-1 sequences have been identified as part of the CRF130_A1B strain, five of which were sampled in Cyprus.

The CRF131_A1B was found in four HIV-1 recombinant viruses sampled in Cyprus. All four of these recombinants exhibited an identical intersubtype mosaic structure. Similar to that of the CRF130_A1B strain, this distinctive configuration consisted of eight distinct fragments separated by seven intersubtype recombination breakpoints. The intersubtype mosaic pattern of this strain was a result of recombination events between the A1 and B lineages, uniting genetic elements from these two lineages forming a distinctive structure specific to CRF131_A1B. 

Regarding the clinical, epidemiological, behavioral, and demographic information, the CRF131_A1B putative transmission cluster was all male, with the majority of them indicating Cyprus as their country of origin. Among the putative transmission cluster, half of which were MSM, there was also one homo/bisexual and one heterosexual individual, indicating the potential spread of this strain to the heterosexual community. Half of the participants lived in urban Nicosia, while one resided in Larnaca and another in Paralimni, showing that the CRF131_A1B strain is mostly circulating in the major epicenter of the HIV-1 epidemic, with the potential to spread to the second largest epicenter, Larnaca. During the BLAST analyses, we did not discover any HIV-1 sequences with a recombinant mosaic structure identical to that of the newly identified CRF131_A1B strain. This observation lends weight to the hypothesis that this recombinant strain originated on the island. However, to gain a more precise understanding of where and when the CRF131_A1B strain originated, it is essential to conduct a more in-depth analysis of spatiotemporal evolutionary dynamics.

Applying the aforementioned criteria, it becomes apparent that the CRF131_A1B putative transmission cluster retains its activity, as the most recent entry into this cluster was recorded in July 2020. Following the characterization of the CRF131_A1B strain, four more HIV-1 sequences exhibiting the same recombinant mosaicism were added to this recombinant putative transmission cluster in December 2021, July 2022, September 2022, and November 2022. This reflects a significant increase in the size of this recombinant putative transmission cluster size over a short period. These strains were isolated from four male individuals, namely, CY869, CY954, CY989, and CY1002, the majority of whom identified as MSM and one as homo/bisexual; all of them cited Cyprus as both their country of origin and infection. An intriguing observation emerged when considering their residences, with three out of the four individuals reporting Larnaca as their home, while one resided in Nicosia. This observation corroborates our earlier prediction regarding the expansion of the CRF131_A1B strain toward the second major epicenter. As a result, currently, there are eight HIV-1 sequences belonging to the CRF131_A1B strain, all sampled in Cyprus.

In conclusion, the CRF138_cpx was identified in not only four HIV-1 recombinant viruses from Cyprus but also two HIV-1 recombinant viruses from Belgium and one from Cameroon, as identified through BLAST analyses. All seven of these HIV-1 recombinant sequences demonstrated uniform intersubtype mosaicism. The mosaic pattern of CRF138_cpx is marked by the presence of six fragments divided by five intersubtype recombination breakpoints. Each of the identified fragments originated from both the CRF22_01A1 and F2 lineages, alongside an additional fragment with an unknown subtype origin, which had not been previously characterized. This synthesis combines elements from three distinct lineages into a novel mosaic arrangement. 

The analysis of clinical, epidemiological, behavioral, and demographic information has provided intriguing insights into the composition of the CRF138_cpx putative transmission cluster. This particular cluster, like the other three novel CRFs, was exclusively detected in male individuals, all of whom reported contracting the virus within Cyprus. Furthermore, three of the four individuals in this cluster originated from Cyprus; nonetheless, one individual reported his country of origin as Cameroon. Additionally, three of these individuals were living in Nicosia, while one was based in Larnaca at the time of sampling, the two epicenters of the HIV-1 epidemic in Cyprus. This scenario suggests the possibility of this novel CRF spreading to the second epicenter of the HIV-1 epidemic, Larnaca, potentially leading to expansion of the cluster in the future. This cluster is associated with a diversity of risk groups, with two individuals self-identifying as MSM, one as heterosexual, and one as both an IDU and a member of the MSM community. This intriguing combination of risk groups raises important questions about potential modes of transmission. Specifically, the presence of an IDU within the cluster suggests the possible transmission of the CRF138_cpx strain within this group, warranting a more in-depth investigation into this possibility. The inclusion of an IDU further emphasizes the need for comprehensive public health measures to address diverse transmission routes, ensuring that prevention and intervention strategies effectively target all at-risk populations via tailored approaches.

In terms of the date of the most recent entry to this putative transmission cluster in September 2021, with a cluster depth of less than or equal to five years, this putative transmission cluster continues to exhibit activity, signifying the most recent growth among the four recently characterized strains. Notably, following the in-depth characterization of the CRF138_cpx strain, an additional HIV-1 sequence exhibiting the same recombinant mosaicism was incorporated into this recombinant putative transmission cluster in March 2022. This sequence was obtained from an individual of the MSM community, CY898, who was residing in Nicosia at the time of sample collection and reported Cyprus as both his country of origin and country of infection, aligning with the prevailing traits of the cluster.

Additionally, the discovery of isolate B (GenBank accession number: MN989925) and isolate EH (GenBank accession number: MT417770), collected in Belgium in September and October 2019, respectively, as well as isolate CHU3903 (GenBank accession number: KP718932), collected in Cameroon in 2011, through BLAST analyses unveiled an intersubtype mosaic structure identical to that of the CRF138_cpx strain [36,37]. This implies that the CRF138_cpx strain has been circulating for some time, as well as the potential introduction of the CRF138_cpx strain from Cameroon to Cyprus. This is supported by the substantial difference in sampling years and the shared country of origin, with the earliest individual in this recombinant putative transmission cluster also originating from Cameroon. However, a more comprehensive investigation of spatiotemporal evolutionary dynamics must be conducted to determine when and where the CRF138_cpx strain originated. Consequently, there are currently eight identified HIV-1 sequences associated with the CRF138_cpx strain, five of which were collected in Cyprus.

As mentioned previously, our diligent exploration based on BLAST analyses unveiled another intriguing isolate that exhibited a noteworthy degree of similarity along with slight differences in its mosaic structure compared to the now well-defined CRF138_cpx strain. Similar to isolate CHU3903, isolate BS72 (GenBank accession number: KR017779) was also collected in Cameroon [38]. A comprehensive examination of the mosaic structure of this sequence, where different subtypes interweave, uncovered the URF of CRF138_cpx. As such, this unique mosaic arrangement consisted of nine discrete fragments separated by eight intersubtype recombination breakpoints. Each of the identified fragments originated from both the CRF22_01A1 and F2 lineages, with two additional fragments of unidentified subtype origin, which had not been previously characterized. 

Comparison between the CRF138_cpx strain and its URF shows that the URF was created by recombination of a fragment belonging to the F2 lineage and another fragment with an unknown subtype origin into the backbone of the newly characterized CRF138_cpx strain. Nevertheless, it was uncovered that this particular sample was collected considerably earlier than the samples associated with the CRF138_cpx strain, dating back to 2007. While the timeline suggests the possibility that the URF predated CRF138_cpx, the precise sequence of events remains unknown, requiring further investigation of the spatiotemporal evolutionary dynamics. However, it is highly plausible that CRF138_cpx and its URF were introduced to Cyprus from Cameroon. Preliminary findings from the prospective molecular epidemiology study (2017–2021) (C. Topcu et al., manuscript in preparation for publication) [21] support this, revealing that approximately 17% of the cohort indicated their origins in Cameroon, portraying the elevated rate of migration from Sub-Saharan Africa. However, evaluating the global prevalence of these novel CRFs, particularly in Sub-Saharan Africa, presents challenges due to the limited availability of molecular data. In particular, the existing sequence data are predominantly derived from partial sequences obtained in molecular epidemiology studies with a primary focus on drug resistance analyses.

## 5. Conclusions

In conclusion, we have characterized four novel CRFs in Cyprus: CRF129_56G composed of CRF56_cpx and subtype G; CRF130_A1B composed of subtypes A1 and B; CRF131_A1B composed of subtypes A1 and B; and CRF138_cpx composed of CRF22_01A1, subtype F2, and an unclassified subtype. The prevalence of these novel CRFs, predominantly among male individuals, especially among Cypriot MSM, and subsequently among HBC individuals, a substantial number of whom contracted the virus in Cyprus, is consistent with the preliminary findings of the prospective molecular epidemiology study (2017–2021) (C. Topcu et al., manuscript in preparation for publication) [21]. These results indicate that these specific cohorts are a driver of the HIV-1 epidemic in Cyprus. This underscores the need for targeted preventive strategies tailored to these cohorts and raises concerns about the potential further spread of these novel CRFs within these specific populations.

The discovery of these four novel CRFs and one URF of CRF138_cpx is evidence of the increasing emergence of novel CRFs and URFs within polyphyletic HIV-1 epidemics. The characterization of these four novel CRFs holds significant relevance for molecular epidemiology studies aimed at identifying additional HIV-1 sequences belonging to these strains in diverse geographic regions. Additionally, this study underscores the unique nature of each of the newly characterized CRF putative transmission clusters, demonstrating the versatility of HIV-1 transmission pathways. The individuals within each cluster display a variety of risk factors and geographical origins, suggesting the complexity of the epidemic in Cyprus. The geographical location of Cyprus at the crossroads of three continents is a key factor contributing to the influx of a wide spectrum of HIV-1 strains and recombinants into the island. This highlights the critical importance of ongoing molecular epidemiology investigations for HIV-1 transmission surveillance. Further in-depth epidemiological studies, complemented by rigorous phylodynamic and phylogeographic analyses, are essential for unraveling the complex spatiotemporal origins of these strains. This, in turn, can provide insights into migration routes and their role in shaping the dynamics of the epidemic. Furthermore, this study underlines the significance of comprehensive analyses involving full- or near-full-length HIV-1 genomes in gaining a deeper understanding of the complex dynamics and diversity of the HIV-1 epidemic, particularly in regions characterized by substantial genetic diversity.

## Figures and Tables

**Table 1:** Clinical, epidemiological, behavioral, and demographic information of the 18 individuals living with HIV-1 included in this study.

Patient ^1^	Sex ^2^	Age Group (Years)	Sample Collection Date	Positive Test Date ^3^	Country of Origin ^4^	Risk Group ^5^	CD4 (cells/mm³)	Plasma HIV-1 RNA (copies × 10^4^/mL)	Epidemiological Information ^6^	GenBank Accession Numbers
CY448	M	40–49	06/17	08/15	Cyprus	MSM	739	3.08	Infected in Cyprus	129_56G.CY448.ON989239
CY512	M	60–69	03/18	03/18	Cyprus	MSM	150	0.03	Infected in Cyprus	129_56G.CY512.OP781327
CY525	M	50–59	05/18	04/18	Cyprus	MSM	124	13.60	Infected in Greece	129_56G.CY525.ON989282
CY526	M	30–39	05/18	04/18	Lebanon	MSM	631	0.70	Infected in Cyprus	129_56G.CY526.ON989283
CY529	M	30–39	06/18	05/18	Greece	HBC	357	7.66	Infected in Greece	129_56G.CY529.ON989285
CY537	M	50–59	07/18	06/18	Cyprus	HC	729	14.60	Infected in Cyprus	129_56G.CY537.ON989289
CY625	M	30–39	08/19	07/19	Cyprus	HBC	406	26.90	Infected in Cyprus	129_56G.CY625.ON989341
CY397	M	30–39	03/17	06/12	Cyprus	HBC	518	5.04	Infected in Cyprus	130_A1B.CY397.OP781326
CY413	M	50–59	03/17	10/13	Cyprus	HBC	1216	2.50	Infected in Cyprus	130_A1B.CY413.ON989226
CY590	M	30–39	02/19	05/12	Cyprus	MSM	229	2.97	Infected in Cyprus	130_A1B.CY590.OP781328
CY584	M	50–59	01/19	01/19	Cyprus	MSM	9	0.38	Infected in Venezuela	131_A1B.CY584.ON989320
CY620	M	40–49	07/19	05/19	Cyprus	HC	450	12.40	Infected in Cyprus	131_A1B.CY620.ON989339
CY697	M	30–39	06/20	04/20	Cyprus	MSM	500	7.31	Infected in Cyprus	131_A1B.CY697.OP781329
CY710	M	40–49	07/20	06/20	Romania	HBC	330	5.51	Infected in Romania	131_A1B.CY710.OP781330
CY639	M	30–39	10/19	08/19	Cameroon	MSM/IDU	388	21.90	Infected in Cyprus	138_CPX.CY639.OP894080
CY805	M	30–39	06/21	05/21	Cyprus	MSM	466	1.46	Infected in Cyprus	138_CPX.CY805.OP894081
CY824	M	50–59	07/21	07/21	Cyprus	MSM	13	900.00	Infected in Cyprus	138_CPX.CY824.OP894082
CY842	M	40–49	09/21	09/21	Cyprus	HC	735	4.87	Infected in Cyprus	138_CPX.CY842.OP894083

^1^ Indicates the unique laboratory identification number, in which the prefix CY is followed by a number denoting the laboratory code. ^2^ M, male. ^3^ Indicates the date (month/year) of the first known positive HIV antibody test. ^4^ Country of birth of the study participants. ^5^ HBC, homo/bisexual contact; MSM, men who have sex with men; HC, heterosexual contact; IDU, injecting drug user. ^6^ Information provided by the study participants with regards to the country where HIV-1 infection occurred.

**Table 2:** Clinical status, antiretroviral treatment status, and drug resistance mutations of the 18 individuals living with HIV-1 included in this study.

Patient ^1^	Clinical Status	Antiretroviral Treatment Status	Drug Resistance Mutations	
			Accessory Drug Resistance Mutations ^2^	Major Drug Resistance Mutations ^3^
CY448	Chronically Infected	No	L10I, K20I, L74I	
CY512	Newly Diagnosed	No	L10I, K20I, L74I	
CY525	Newly Diagnosed	No		
CY526	Newly Diagnosed	No		
CY529	Newly Diagnosed	No		
CY537	Newly Diagnosed	No	L10I, K20I, L74I	
CY625	Newly Diagnosed	No	L10I, K20I, L74I	
CY397	Chronically Infected	Yes		
CY413	Chronically Infected	No	A71V	L33F
CY590	Chronically Infected	Yes		
CY584	Newly Diagnosed	No	A71V	
CY620	Newly Diagnosed	No	A71V	E138A
CY697	Newly Diagnosed	No	A71V	E138A
CY710	Newly Diagnosed	No	A71V	E138A
CY639	Newly Diagnosed	No		
CY805	Newly Diagnosed	No	L10I, K20R	
CY824	Newly Diagnosed	No	L10I, K20R	
CY842	Newly Diagnosed	No	L10I, K20R	

^1^ Indicates the unique laboratory identification number, in which the prefix CY is followed by a number denoting the laboratory code. ^2^ Accessory drug resistance mutations refer to genetic alterations in the HIV genome that might arise due to the selective pressure exerted by antiretroviral drugs. These mutations, when occurring independently, may not exert as substantial an impact on drug susceptibility as major drug resistance mutations. ^3^ Major drug resistance mutations involve genetic modifications in the HIV genome that are linked to a notable decrease in the virus’s susceptibility to one or more antiretroviral drugs.

## Data Availability

The near-full-length- and partial HIV-1 genome nucleotide sequence data of the novel recombinant viruses generated in this study are openly available in GenBank at https://www.ncbi.nlm.nih.gov/genbank/ (accessed on 13 November 2023), and the accession numbers are ON989239, OP781327, ON989282, ON989283, ON989285, ON989289, ON989341, OP781326, ON989226, OP781328, ON989320, ON989339, OP781329, OP781330, OP894080, OP894081, OP894082, and OP894083. The GenBank accession numbers of the top BLAST hit sequences, which exhibited intersubtype mosaicism identical to CRF130_A1B and CRF138_cpx, were MW063005, and MN989925, MT417770, and KP718932, respectively. The GenBank accession number for the URF of CRF138_cpx, showing intersubtype mosaicism similar to CRF138_cpx with slight differences, was KR017779. The GenBank accession numbers of the sequences used in the reference dataset for the bootscan, similarity plot, and the confirmatory phylogenetic tree analyses of subregions for each of the four novel CRFs were as follows. For CRF129_56G: A1-AF004885, A1-AF069670, A1-U51190, A1-AF484509; A2-AF286238, A2-GU201516, A2-AF286237; A3-AY521629, A3-AY521630, A3-AY521631; A4-AM000053, A4-AM000054; A6-KT983615, A6-AF413987, A6-DQ207944, A6-EU861977; A7-KX389622, A7-KX389608, A7-MH078558; A8-MW353966, A8-MW353967; B-K03455, B-AY173951, B-AY423381, B-AY331295; C-U52953, C-U46016, C-AF067155, C-AY772699; D-K03454, D-U88824, D-AY253311, D-AY371157; F1-AF077336, F1-AF005494, F1-AF075703, F1-AJ249238; F2-AY371158, F2-AF377956, F2-AJ249237, F2-AJ249236; G-AF084936, G-U88826, G-AF061642, G-AF061641; H-FJ711703, H-AF190127, H-AF190128, H-AF005496; J-GU237072, J-AF082395, J-AF082394; K-AJ249235, K-AJ249239; L-MN271384, L-AF286236, L-AF457101; CRF56_cpx-JN882655, CRF56_cpx-KC852172, CRF56_cpx-KC852173, and CRF56_cpx-KC852174. For both CRF130_A1B and CRF131_A1B: A1-AF004885, A1-AF069670, A1-U51190, A1-AF484509; A2-AF286238, A2-GU201516, A2-AF286237; B-K03455, B-AY173951, B-AY423381, B-AY331295, B-DQ076814, B-JX147100, B-DQ076812, B-GU330270, B-GU330253, B-KM355143, B-KT339988, B-KM355099, B-KM355145, B-KX129256, B-KM355092, B-JF683781, B-KM355154, B-KM355116, B-KM355146, B-KM355144, B-KM355149, B-JF683781; C-U52953, C-U46016, C-AF067155, C-AY772699; D-K03454, D-U88824, D-AY253311, D-AY371157; F1-AF077336, F1-AF005494, F1-AF075703, F1-AJ249238; F2-AY371158, F2-AF377956, F2-AJ249237, F2-AJ249236; G-AF084936, G-U88826, G-AF061642, G-AF061641; H-FJ711703, H-AF190127, H-AF190128, H-AF005496; J-GU237072, J-AF082395, J-AF082394; K-AJ249235, K-AJ249239; L-MN271384, L-AF286236, and L-AF457101. For bootscan and similarity plot for CRF138_cpx: B-K03455, B-AY173951, B-AY423381, B-AY331295; C-U52953, C-U46016, C-AF067155, C-AY772699; D-K03454, D-U88824, D-AY253311, D-AY371157; F2-AY371158, F2-AJ249237, F2-AJ249236, F2-KR822828; H-FJ711703, H-AF190127, H-AF190128, H-AF005496; J-GU237072, J-AF082395, J-AF082394; K-AJ249239; L-AF457101; CRF22_01A1-KF716460, CRF22_01A1-KX228816, CRF22_01A1-KF716462, CRF22_01A1-KP109499, and CRF22_01A1-JN864050. For confirmatory phylogenetic tree analyses of subregions for CRF138_cpx: B-K03455, B-AY173951, B-AY423381, B-AY331295; C-U52953, C-U46016, C-AF067155, C-AY772699; D-K03454, D-U88824, D-AY253311, D-AY371157; F2-AY371158, F2-AF377956, F2-AJ249237, F2-AJ249236; G-AF084936, G-U88826, G-AF061642, G-AF061641; H-FJ711703, H-AF190127, H-AF190128, H-AF005496; J-GU237072, J-AF082395, J-AF082394; K-AJ249235, K-AJ249239; L-MN271384, L-AF286236, L-AF457101; and CRF22_01A1-AY371159.

## Supplementary Materials

The following supporting information can be downloaded at https://www.mdpi.com/article/10.3390/v16010019/s1, Figure S1: Recombination analyses of the 16 near-full-length HIV-1 genome (790–8795 in the HXB2 genome) nucleotide sequences derived from samples CY448, CY512, CY526, CY529, CY537, CY625, CY413, 5112_MW063005, CY584, CY620, CY697, CY805, CY824, B_MN989925, EH_MT417770, and CHU3903_KP718932, and two HIV-1 partial genome nucleotide sequences derived from samples CY590 (790–5250 in the HXB2 genome) and CY842 (2253–8795 in the HXB2 genome) illustrating the unique intersubtype mosaic structure of the CRF129_56G, CRF130_A1B, CRF131_A1B, and CRF138_cpx strains; Figure S2: Comparison of the intersubtype recombination breakpoints between CRF130_A1B and CRF131_A1B strains; Figure S3: Recombination analyses of isolate 5112 (GenBank accession number: MW063005) using two different reference datasets; Figure S4: Recombination analyses of the near-full-length HIV-1 genome (790–8795 in the HXB2 genome) nucleotide sequence of isolate BS72 (GenBank accession number: KR017779) used to illustrate the unique intersubtype mosaic structure of the URF of the CRF138_cpx strain, “Rec. of 138_cpx, F2”; Figure S5: Recombination analyses of isolate BS72 (GenBank accession number: KR017779) using two different reference datasets. References [14,22,25,26,27,28,30,31,34] are cited in the Supplementary Materials.
