# Supplementary material for: Comprehensive Genetic Characterization of Four Novel HIV-1 Circulating Recombinant Forms (CRF129_56G, CRF130_A1B, CRF131_A1B, and CRF138_cpx): Insights from Molecular Epidemiology in Cyprus
Source: Viruses. 2023 Dec 21;16(1):19. doi: 10.3390/v16010019 (PMC10819958; doi:10.3390/v16010019)
Supplement: Supplementary file 1 [file viruses-16-00019-s001.zip › viruses-2746542-supplementary.pdf]

Sample CY448

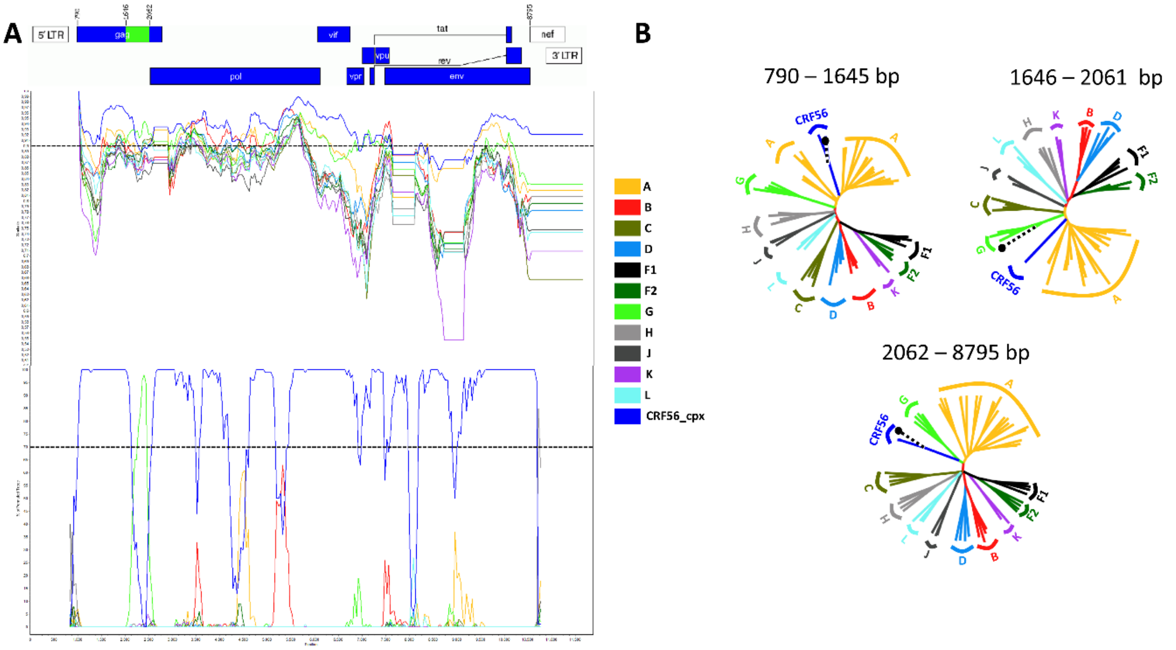

Sample CY512

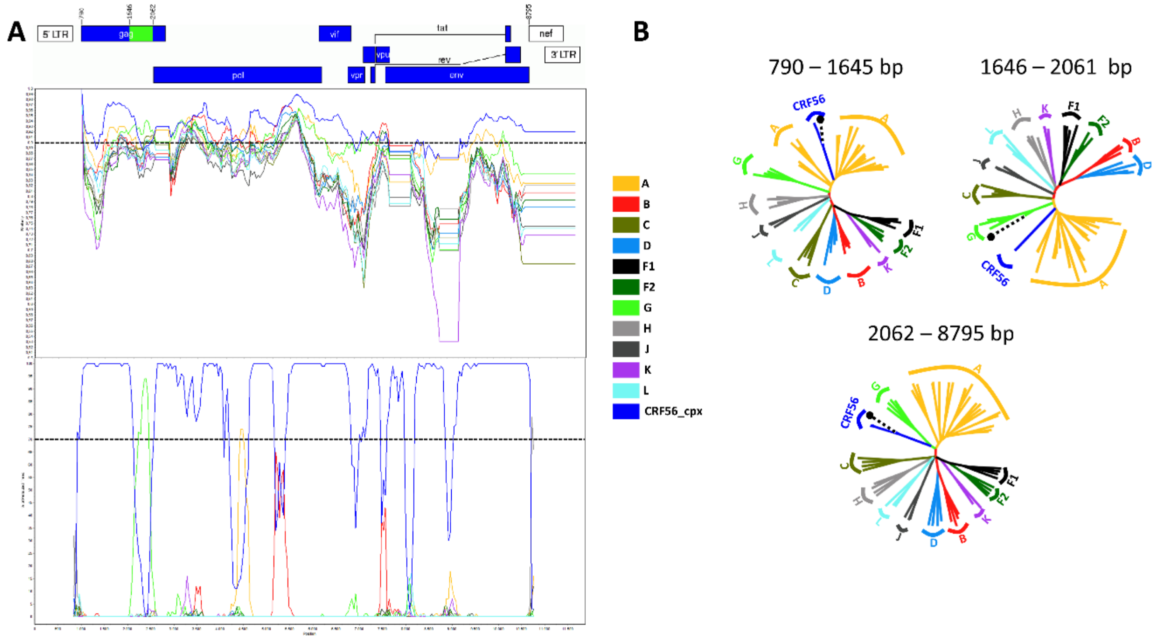

Sample CY526

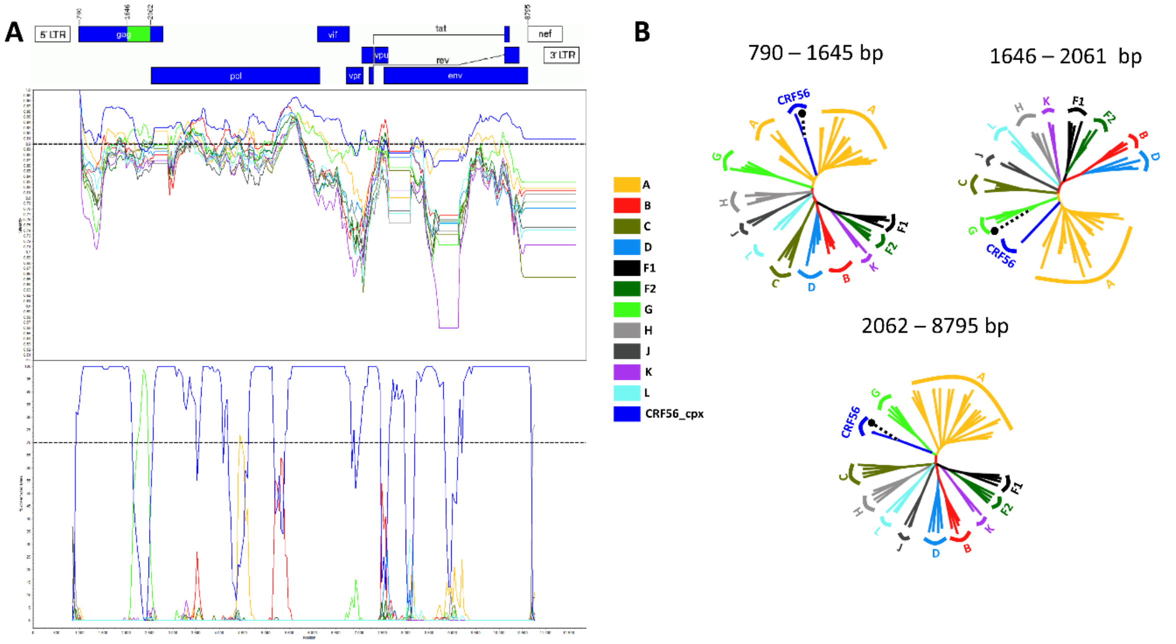

Sample CY529

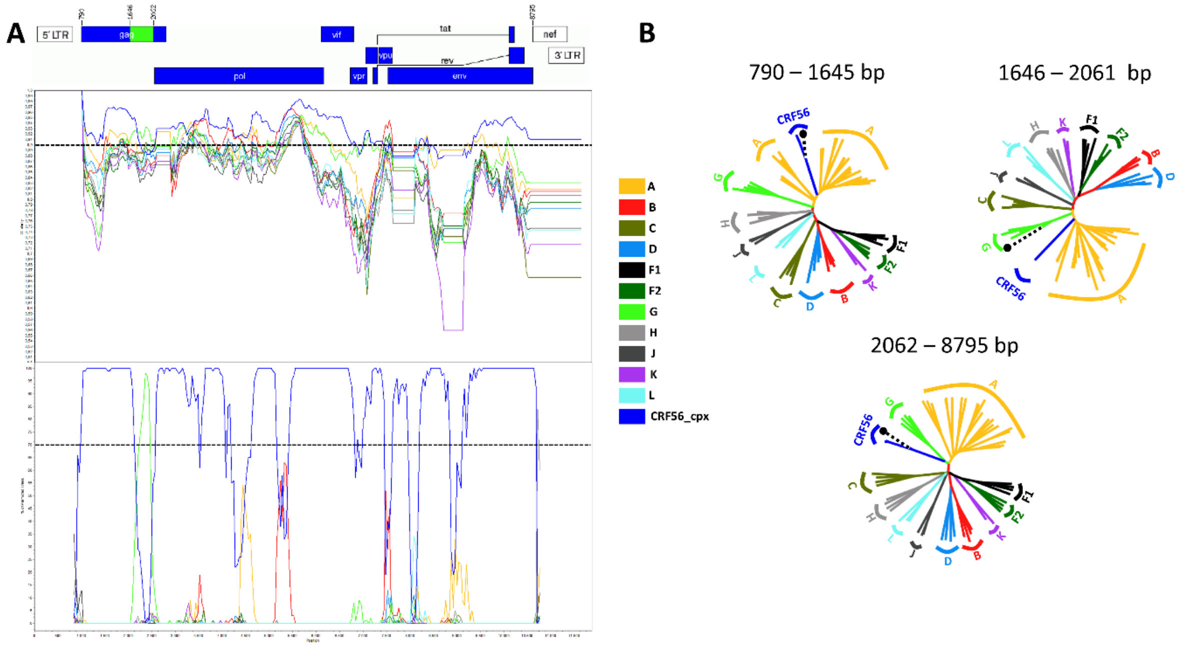

Sample CY537

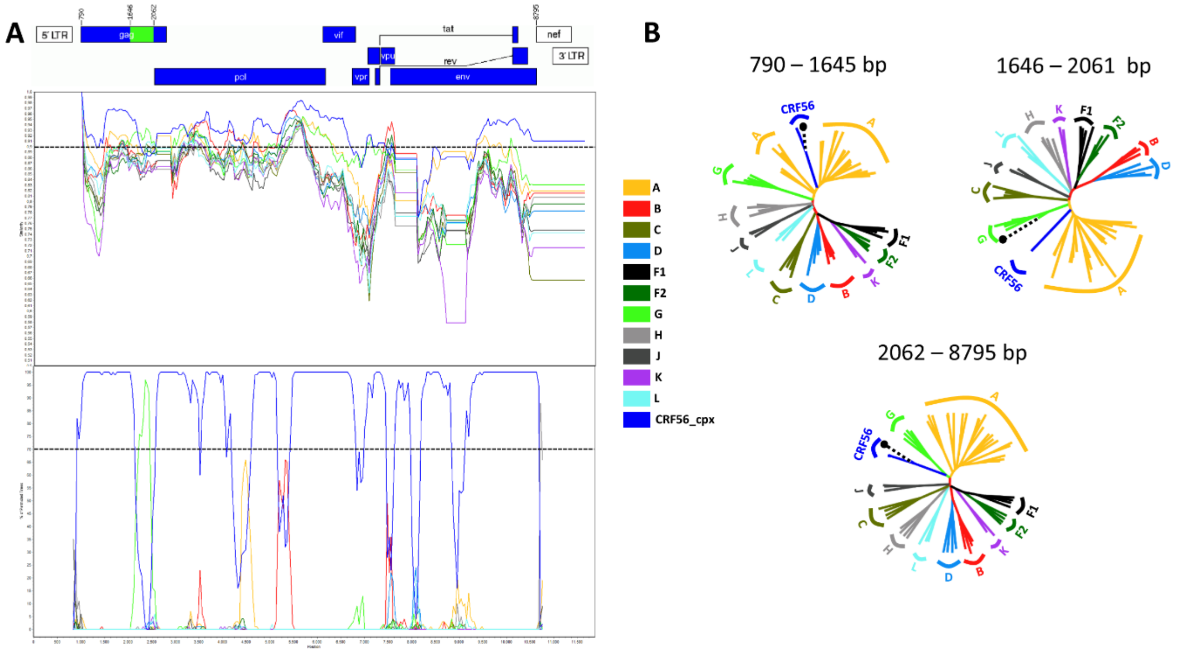

Sample CY625

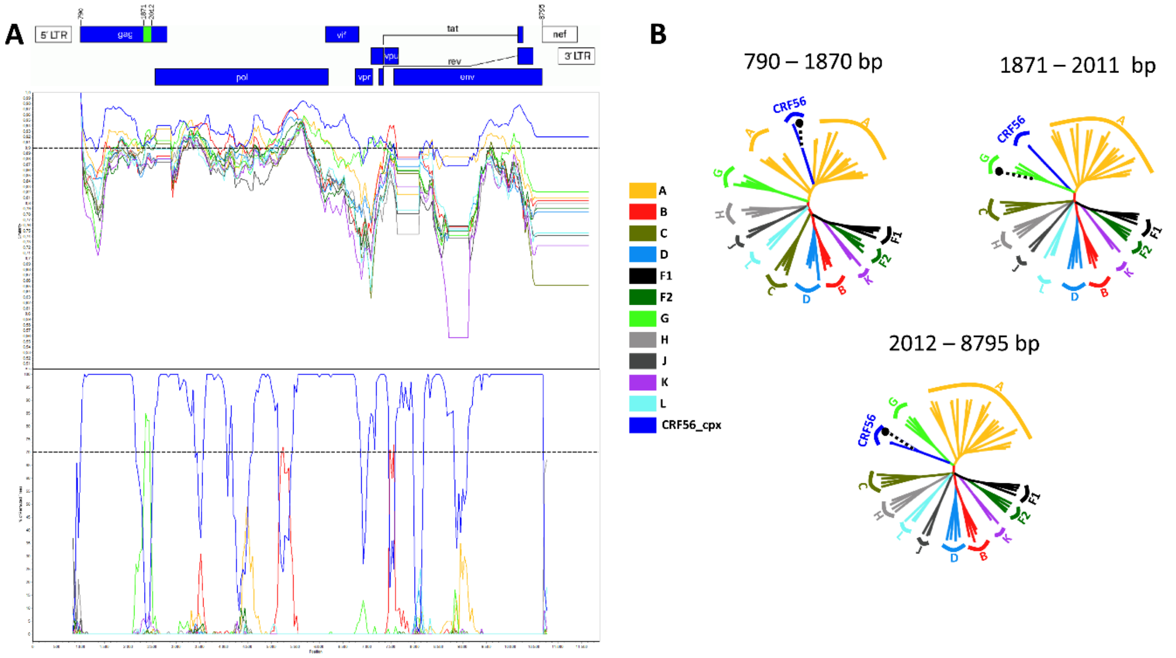

Sample CY413

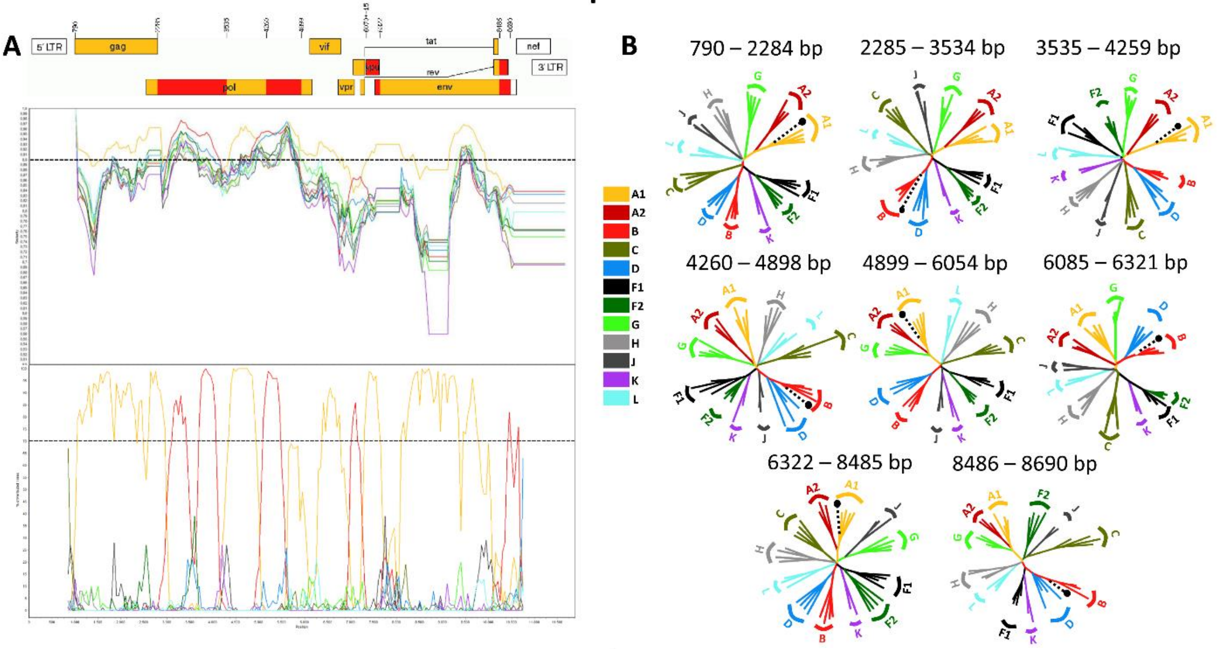

Sample CY590

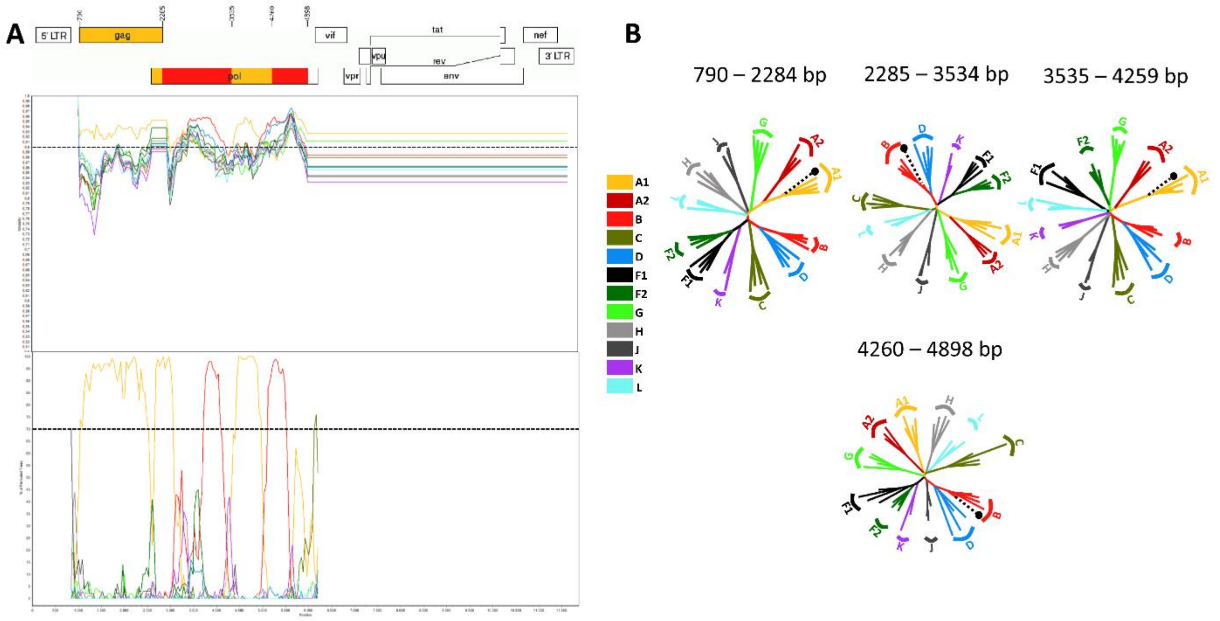

Sample 5112_MW063005 (USA)

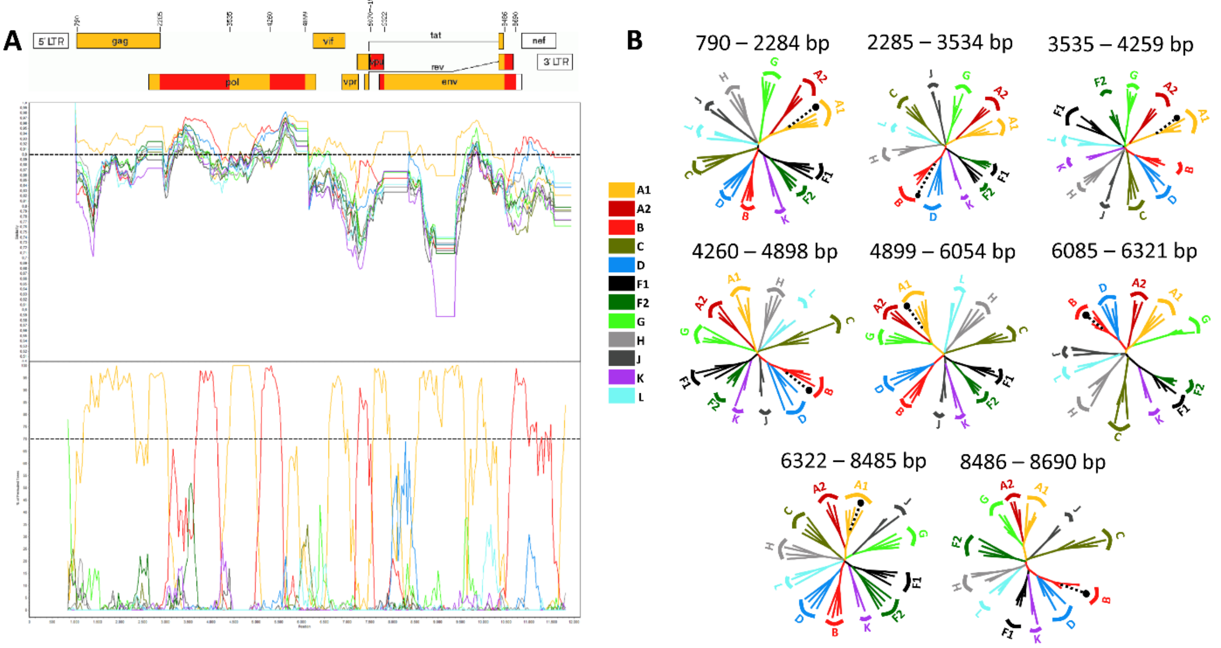

Sample CY584

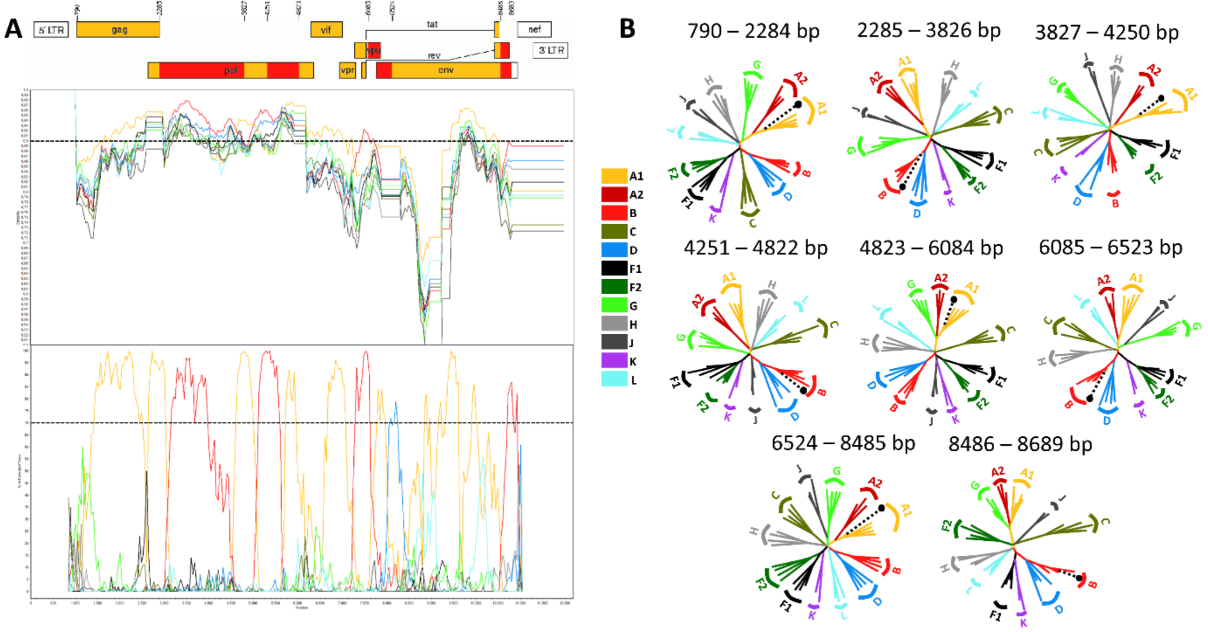

Sample CY620

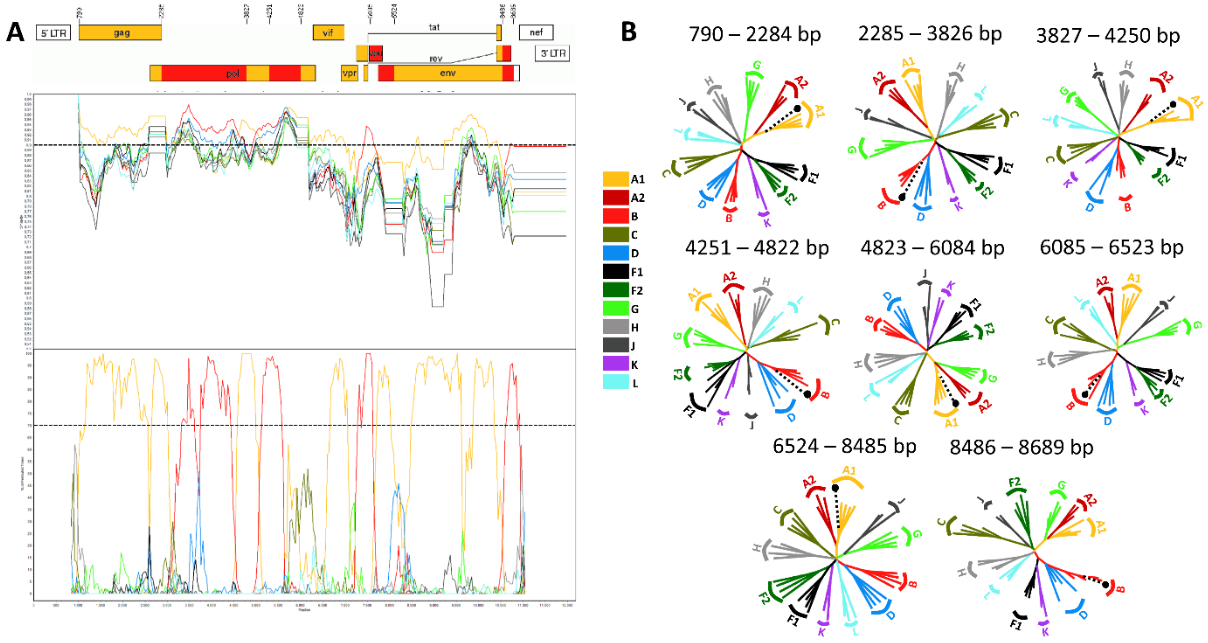

Sample CY697

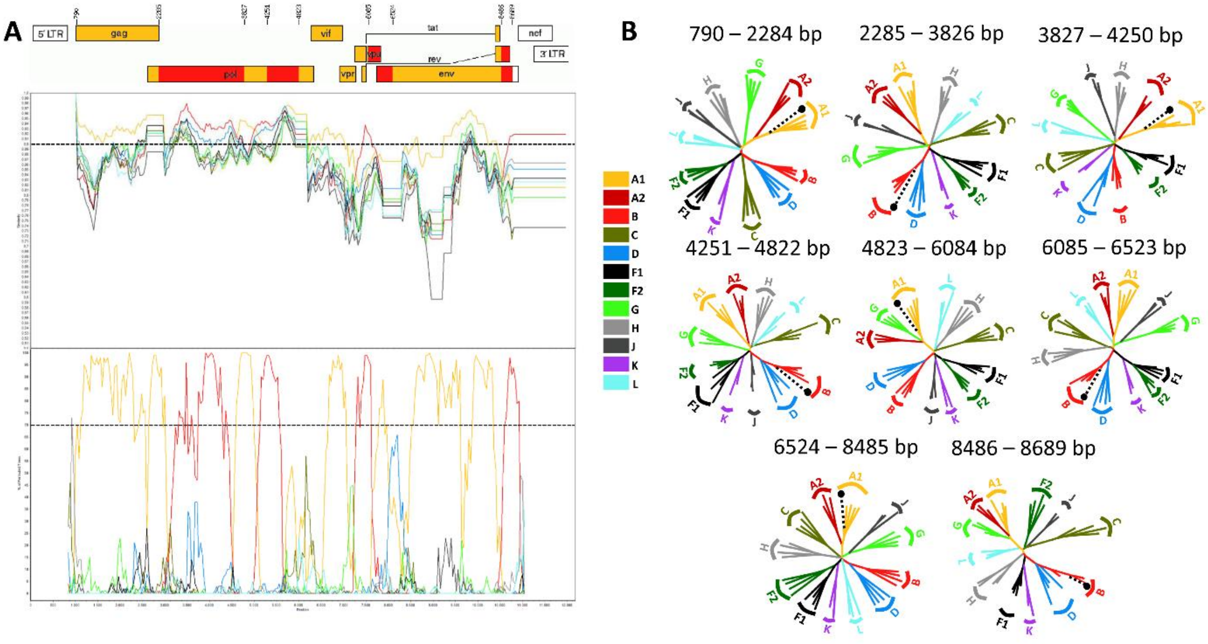

Sample CY805

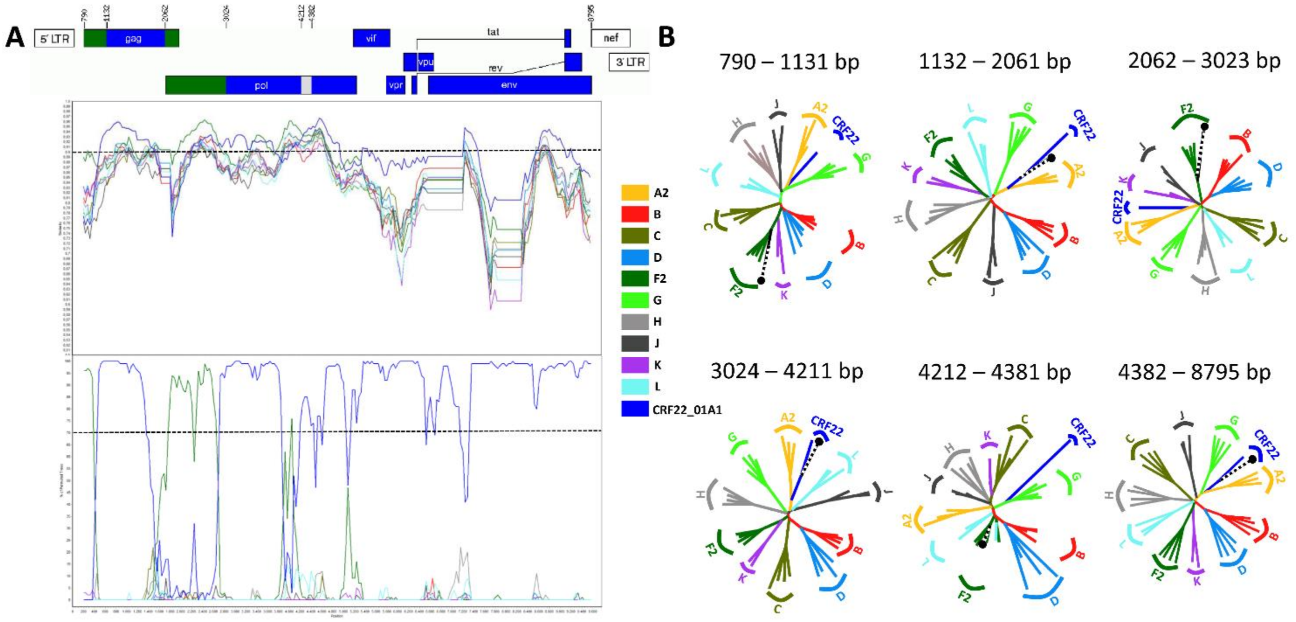

Sample CY824

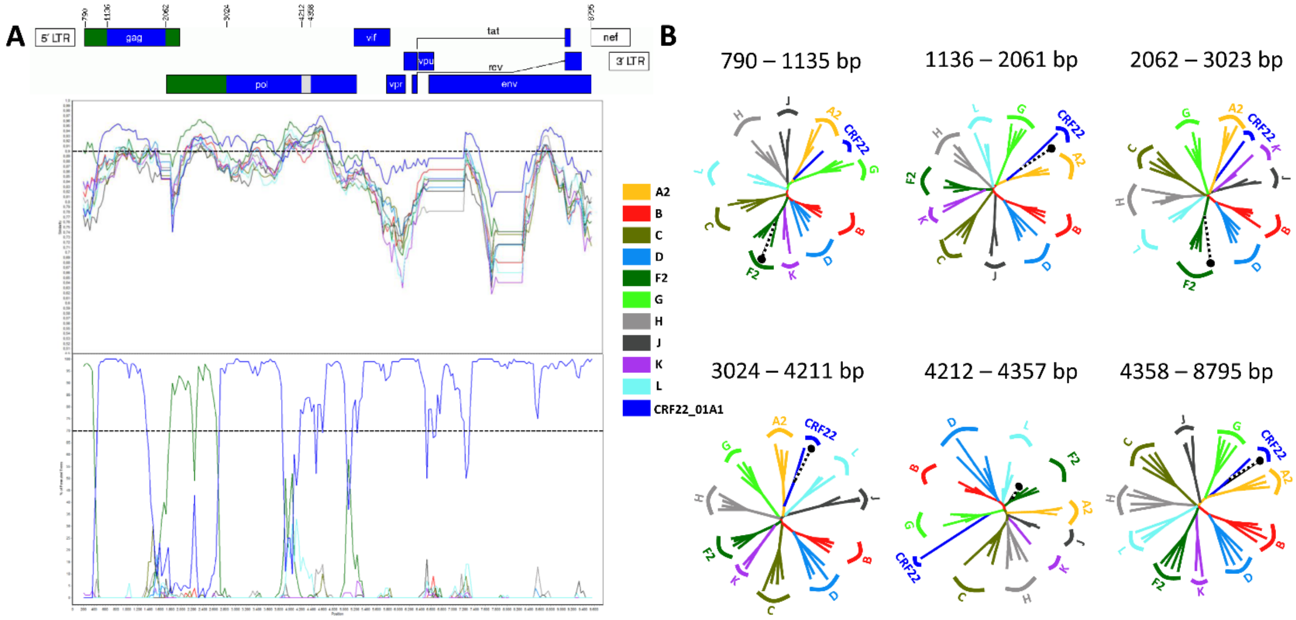

Sample CY842

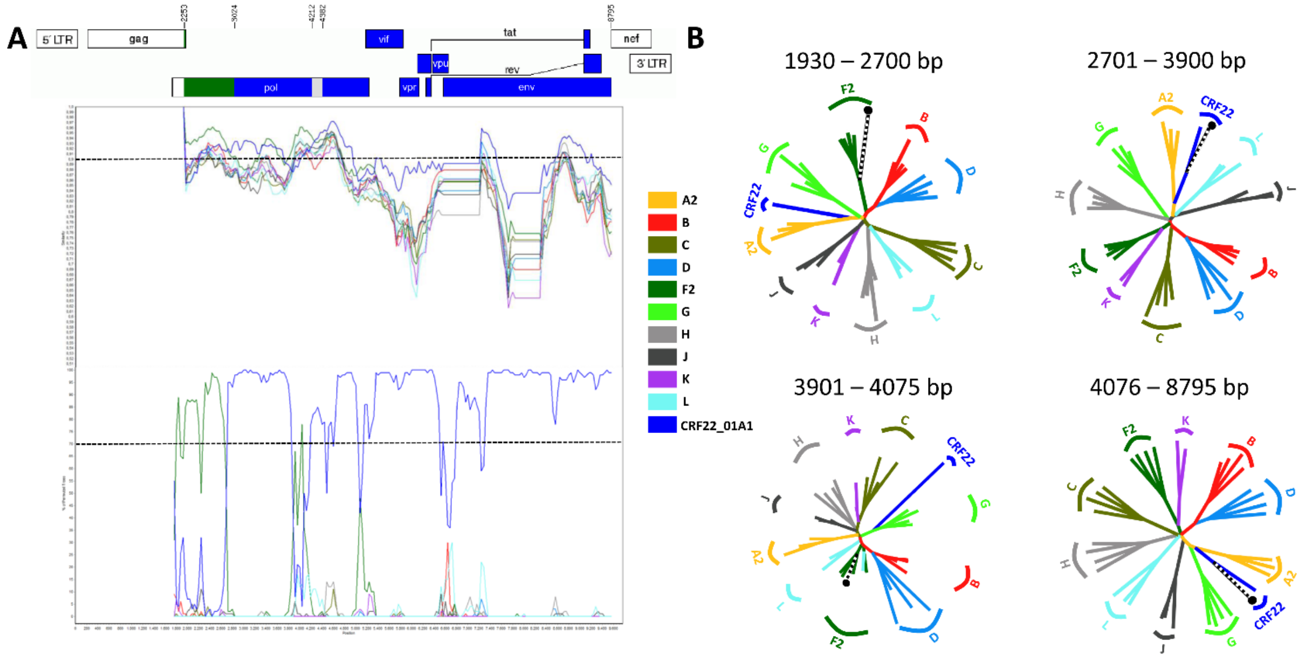

Sample B_MN989925 (Belgium)

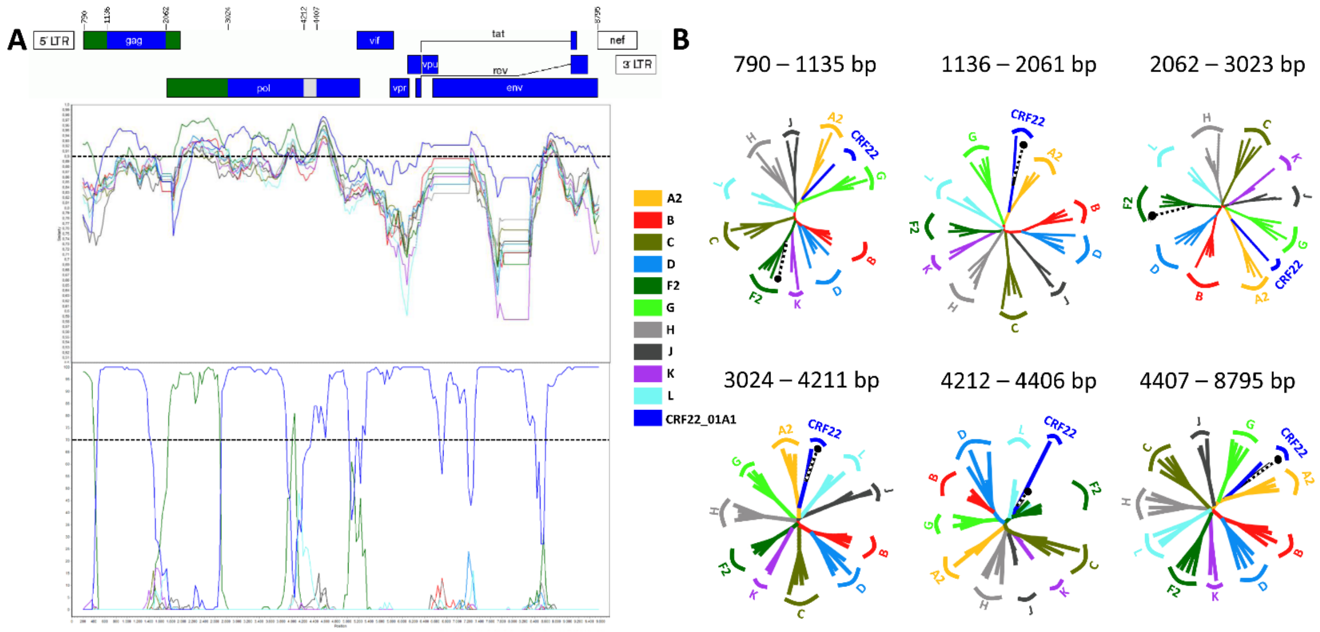

Sample EH_MT417770 (Belgium)

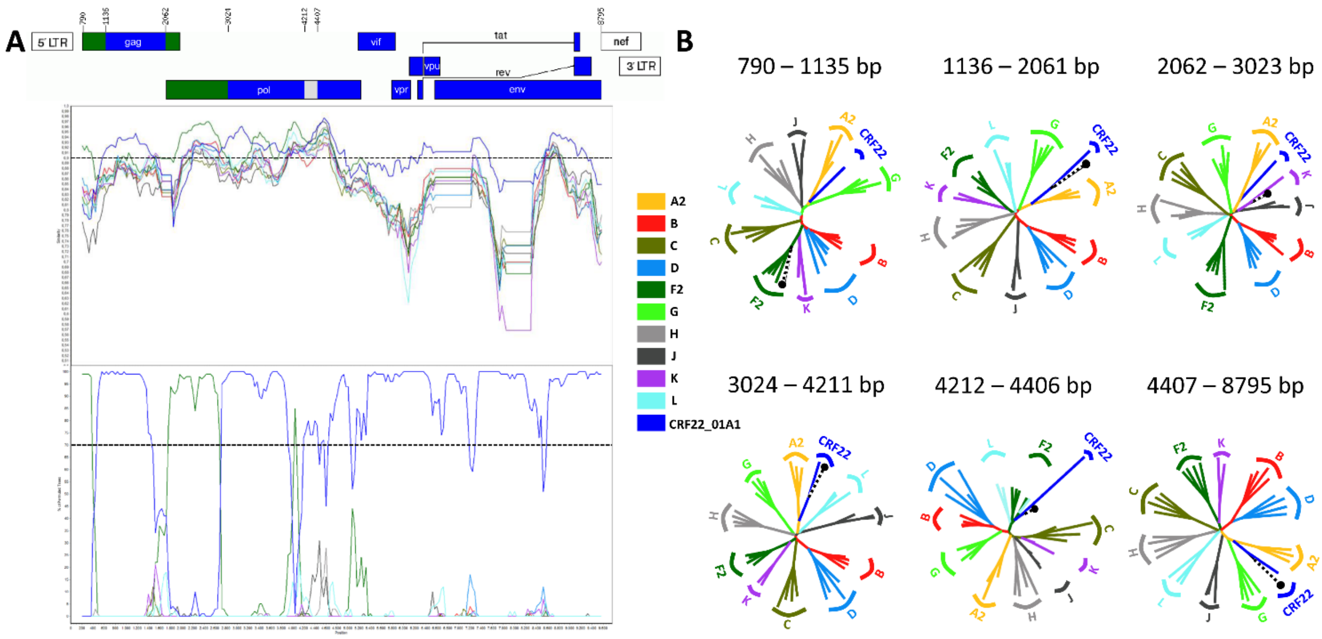

Sample CHU3903_KP718932 (Cameroon)

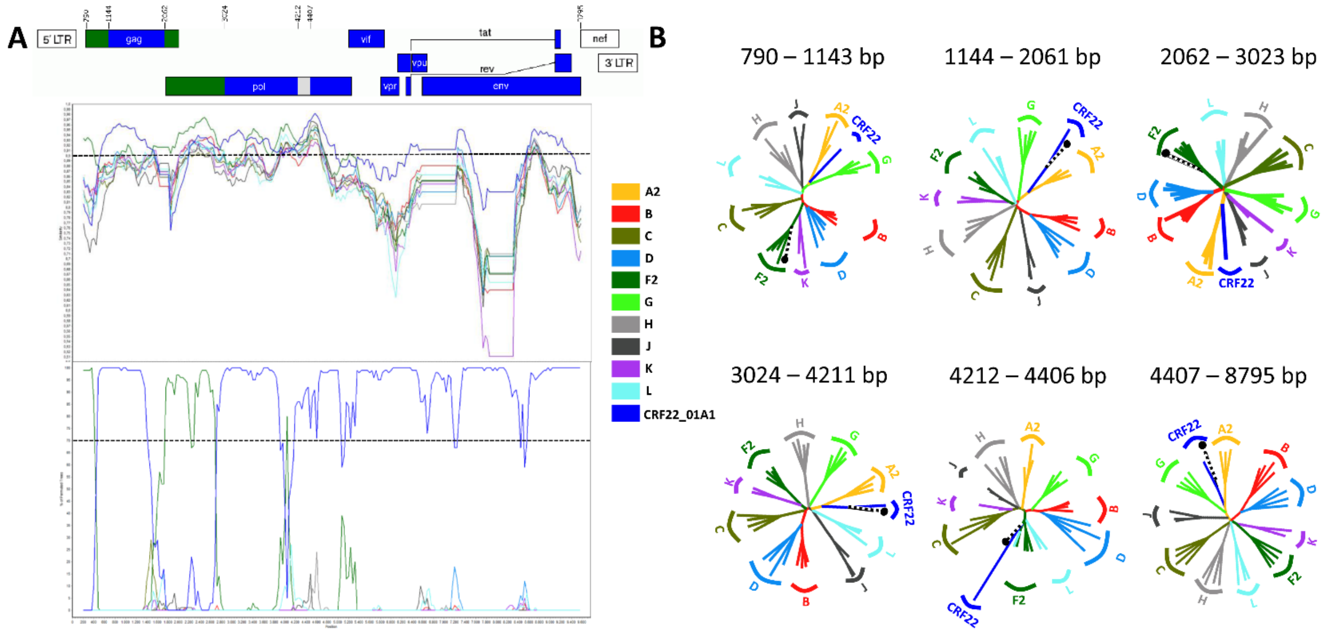

Figure S1: Recombination analyses of the 16 near-full-length HIV-1 genome (790-8795 in the HXB2 genome) nucleotide sequence derived from samples CY448, CY512, CY526, CY529, CY537, CY625, CY413, 5112_MW063005, CY584, CY620, CY697, CY805, CY824, B_MN989925, EH_MT417770, and CHU3903_KP718932, and two HIV-1 partial genome nucleotide sequences derived from samples CY590 (790-5250 in the HXB2 genome) and CY842 (2253-8795 in the HXB2 genome) illustrating the unique intersubtype mosaic structure of the CRF129_56G, CRF130_A1B, CRF131_A1B and CRF138_cpx strains. Each scheme in this figure characterizes each of the aforementioned samples, as denoted above each scheme. The recombination and phylogenetic analyses were conducted against a reference dataset of HIV-1 group M subtypes (A, B, C, D, F, G, H, J, K, and L), CRF22_01A1 and CRF56_cpx downloaded from the Los Alamos HIV Sequence Database (http://www.hiv.lanl.gov), which was enriched through Basic Local Alignment Search Tool (BLAST) analyses (https://www.hiv.lanl.gov/content/sequence/BASIC_BLAST/basic_blast.html) with the top BLAST hits for subtype B, sub-subtype F2, CRF22_01A1 and CRF56_cpx strains. MEGA X software was employed for the construction of the multiple sequence alignment of the query sequences and the reference dataset using the Clustal W algorithm [1,2]. AliView software was used to visualize and manually edit the multiple sequence alignment [3]. The finalized multiple sequence alignment was utilized for the following recombination and phylogenetic analyses. (**A**) The upper left illustration in each scheme demonstrates the genomic map, as created using the Recombinant HIV-1 Drawing Tool (https://www.hiv.lanl.gov/content/sequence/DRAW_CRF/recom_mapper.html). The numbers above the illustration denote the intersubtype recombination breakpoints with respect to the HXB2 genome. The first six schemes illustrate the intersubtype mosaic pattern of the CRF129_56G strain, in which the near full-length HIV-1 genome was divided into three fragments based on the two recombination breakpoints. The next three schemes illustrate the intersubtype mosaic pattern of the CRF130_A1B strain, in which the near full-length HIV-1 genome was divided into eight fragments based on the seven recombination breakpoints. The next three schemes illustrate the intersubtype mosaic pattern of the CRF131_A1B strain, in which the near full-length HIV-1 genome was divided into eight fragments based on the seven recombination breakpoints. To conclude, the last six schemes illustrate the intersubtype mosaic pattern of the CRF138_cpx strain, in which the near-full-length HIV-1 genome was divided into six fragments based on the five recombination breakpoints. The subtype origin of each fragment is color coded in agreement with all instructive recombination analyses within each scheme, and the color coding is described in the middle of each scheme. The middle left illustration in each scheme presents the similarity plot analysis, where the y-axis shows the percent similarity of the query sequence to the reference dataset. The intermittent horizontal line on the similarity plot depicts the 90% similarity. The lower left illustration in each scheme presents the bootscan analysis, in which the y-axis shows the bootstrap support value. The intermittent horizontal line on the bootscan depicts the threshold of 70% bootstrap support value that was decided to be definitive for subtype origin of each fragment. The x-axes in both illustrations signify the loci with respect to the HXB2 genome. SimPlot v3.5.1 software was used to conduct the similarity plot and bootscan analyses, where a sliding window of 400 nucleotides, overlapped by 40 nucleotides, with 1000 bootstrap replicates were adopted as the optimal parameters [4]. (**B**) The illustration on the right side of each scheme displays the subregion confirmatory neighbor-joining tree analyses. MEGA X software was used to construct neighbor-joining trees for each of the fragments as defined by the prior similarity plot and bootscan analyses within each scheme [1]. Kimura two-parameter nucleotide

substitution model with 1000 bootstrap replicates, which was used to evaluate the reliability of the phylogenetic clustering, were adopted as the optimal parameters for the phylogenetic analyses [5]. Threshold of 70% bootstrap support value was decided to be definitive for subtype origin of each fragment. The loci of the beginning and end of each fragment in accordance with the HXB2 numbering are indicated above each respective phylogenetic tree. The branch displayed by a black intermittent line ending in a black dot demonstrates the query sequence. The color coding of the phylogenetic trees agrees with rest of the recombination analyses within each scheme.

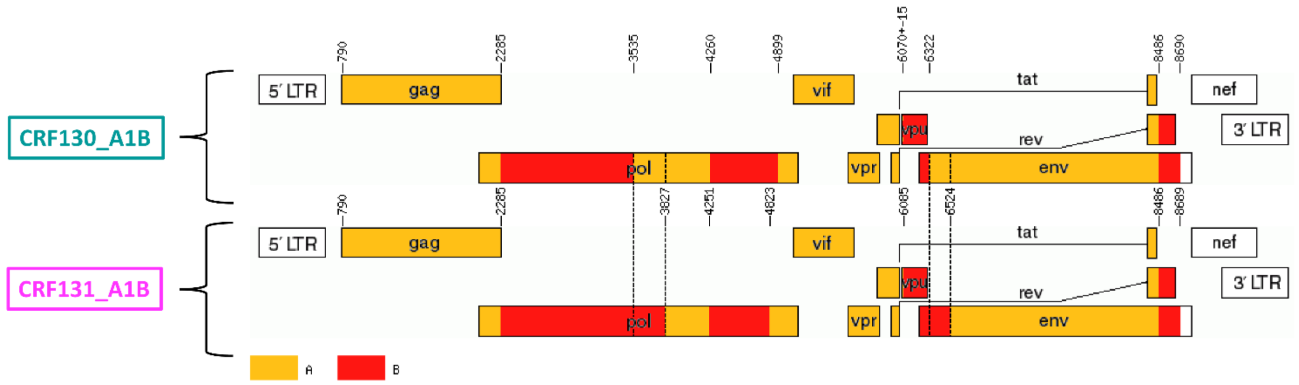

Figure S2: Comparison of the intersubtype recombination breakpoints between CRF130_A1B and CRF131_A1B strains. The upper and lower illustrations demonstrate the consensus genomic maps of the near-full-length HIV-1 genome of CRF130_A1B and CRF131_A1B strains, respectively, as created using the Recombinant HIV-1 Drawing Tool (https://www.hiv.lanl.gov/content/sequence/DRAW_CRF/recom_mapper.html). The numbers above each illustration denote the intersubtype recombination breakpoints with respect to the HXB2 genome as defined by previous instructive recombination analyses. The intermittent vertical lines highlight the differences in recombination breakpoints between the two lineages. Only the differences that are more than 200 nucleotides in length are denoted. The subtype origin of each fragment is color coded in agreement for both illustrations and the color coding is described at the bottom left of the figure.

Sample 5112_MW063005 (USA)

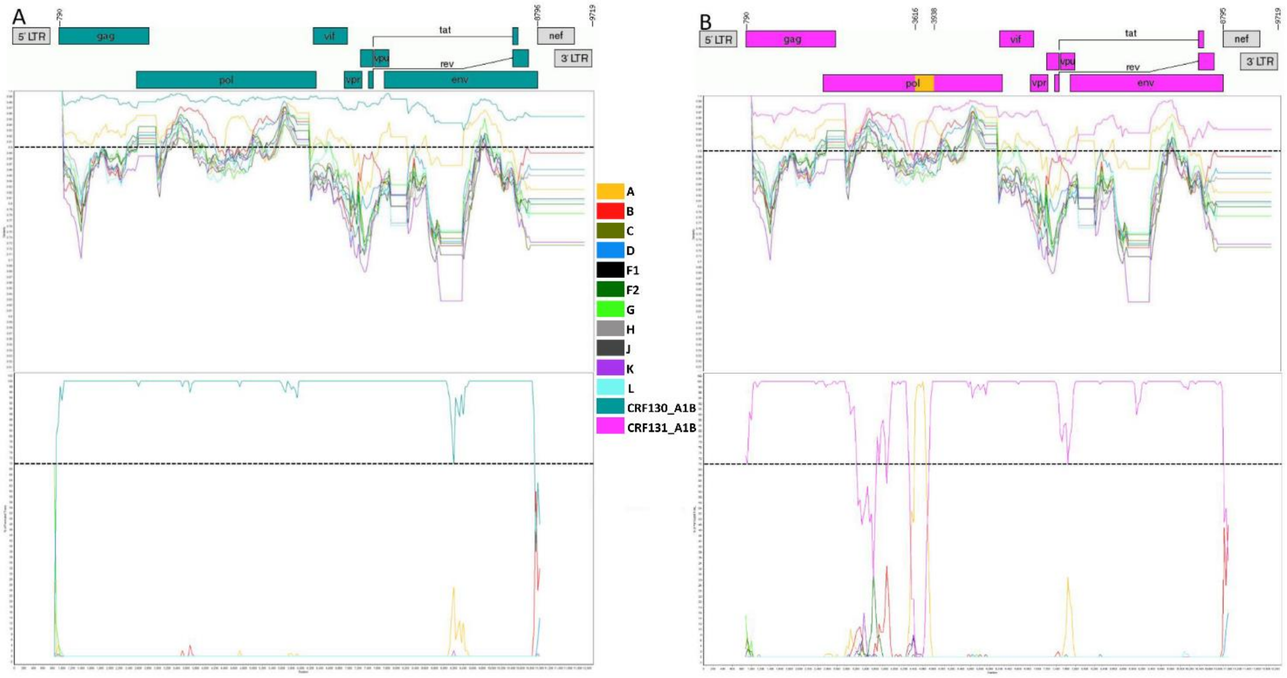

Figure S3: Recombination analyses of isolate 5112 (GenBank accession number: MW063005) using two different reference datasets. Isolate 5112 that was sampled in the United States of America (USA), which was identified through BLAST analyses, demonstrate the same mosaic structure as the CRF130_A1B strain. This comparison was executed to emphasize the differences between mosaic genomic structures of the CRF130_A1B and CRF131_A1B strains. The comparison also illustrates correct strain classification of isolate 5112, validating sufficient divergence between CRF130_A1B and CRF131_A1B for strain differentiation, despite high percent similarity between the mosaic genomic structures of the two strains. MEGA X software was employed for the construction of the multiple sequence alignments of the query sequences and the reference datasets using the Clustal W algorithm [1,2]. AliView software was used to visualize and manually edit the multiple sequence alignment [3]. The finalized multiple sequence alignment was utilized for the following recombination analyses. The subtype origin of each fragment is color coded in agreement with all instructive recombination analyses, and the color coding is described in the middle of the figure. (**A**) The recombination analyses shown by the illustrations on the left side of the figure, were run against a reference dataset of HIV-1 group M subtypes (A, B, C, D, F, G, H, J, K, and L) downloaded from the Los Alamos HIV Sequence Database (http://www.hiv.lanl.gov), along with the three samples from Cyprus belonging to the CRF130_A1B strain. The upper left illustration shows the genomic map, as created using the Recombinant HIV-1 Drawing Tool (https://www.hiv.lanl.gov/content/sequence/DRAW_CRF/recom_mapper.html). The middle left illustration presents the similarity plot analysis, where the y-axis shows the percent similarity of the query sequence to the reference dataset. The intermittent horizontal line depicts the 90% similarity. The lower left illustration presents the bootscan analysis, in which the y-axis shows the bootstrap support value. The intermittent horizontal line depicts the threshold of 70% bootstrap support value that was decided to be definitive for subtype origin of each fragment. The x-axes in both illustrations

signify the loci with respect to the HXB2 genome. SimPlot v3.5.1 software was used to conduct the similarity plot and bootscan analyses, where a sliding window of 400 nucleotides, overlapped by 40 nucleotides, with 1000 bootstrap replicates were adopted as the optimal parameters [4]. (**B**) The recombination analyses, shown by the illustrations on the right side of the figure, were run against a reference dataset of HIV-1 group M subtypes (A, B, C, D, F, G, H, J, K, and L) downloaded from the Los Alamos HIV Sequence Database (http://www.hiv.lanl.gov), along with the four samples from Cyprus belonging to the CRF131_A1B strain. The bootscan and similarity plot analyses were repeated using this reference dataset. The recombination analyses in part A present that isolate 5112 belongs to the CRF130_A1B strain, while the recombination analyses in part B show that there is a difference between isolate 5112 and CRF131_A1B strain at loci 3616-3938 (in the HXB2 genome).

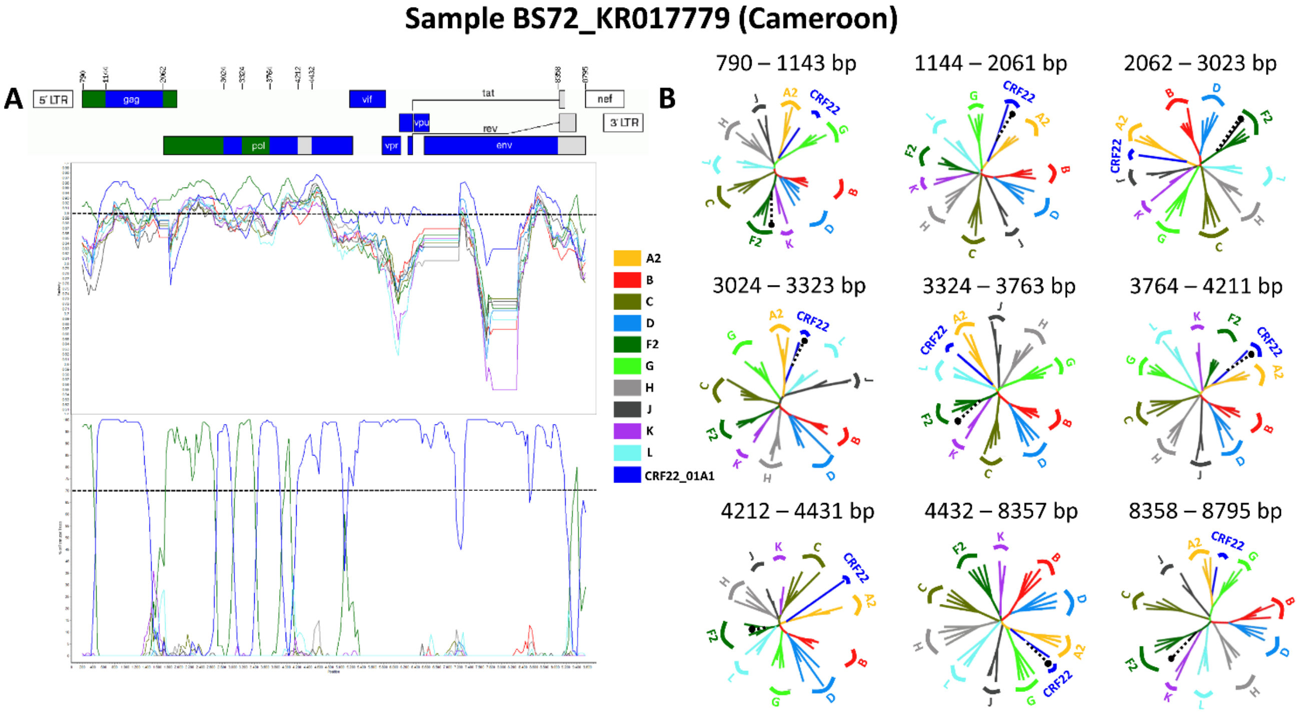

Figure S4: Recombination analyses of the near-full-length HIV-1 genome (790–8795 in the HXB2 genome) nucleotide sequence of isolate BS72 (GenBank accession number: KR017779) used to illustrate the unique intersubtype mosaic structure of the unique recombinant form (URF) of the CRF138_cpx strain, "Rec. of 138_cpx, F2". The isolate BS72, which was discovered through BLAST analyses, demonstrates a very similar yet slightly different mosaic structure to the CRF138_cpx strain. The recombination and phylogenetic analyses were conducted against a reference dataset of HIV-1 group M subtypes (A2, B, C, D, F2, G, H, J, K, and L) and CRF22_01A1 downloaded from the Los Alamos HIV Sequence Database (http://www.hiv.lanl.gov), which was enriched through Basic Local Alignment Search Tool (BLAST) analyses (https://www.hiv.lanl.gov/content/sequence/BASIC_BLAST/basic_blast.html) with six of the top BLAST hits for sub-subtype F2 and CRF22_01A1. MEGA X software was employed for the construction of the multiple sequence alignment of the query sequences and the reference dataset using the Clustal W algorithm [1,2]. AliView software was used to visualize and manually edit the multiple sequence alignment [3]. The finalized multiple sequence alignment was utilized for the following recombination and phylogenetic analyses. (**A**) The upper left illustration demonstrates the genomic map, as created using the Recombinant HIV-1 Drawing Tool (https://www.hiv.lanl.gov/content/sequence/DRAW_CRF/recom_mapper.html). The numbers above the illustration denote the intersubtype recombination breakpoints with respect to the HXB2 genome. The illustration shows the intersubtype mosaic pattern of the URF of CRF138_cpx strain, "Rec. of 138_cpx, F2", in which the near full-length HIV-1 genome was divided into nine fragments based on the eight recombination breakpoints. The subtype origin of each fragment is color coded in agreement with all instructive recombination analyses, and the color coding is described in the middle of the figure. The middle left illustration presents the similarity plot analysis, where the y-axis shows the

percent similarity of the query sequence to the reference dataset. The intermittent horizontal line on the similarity plot depicts the 90% similarity. The lower left illustration presents the bootscan analysis, in which the y-axis shows the bootstrap support value. The intermittent horizontal line on the bootscan depicts the threshold of 70% bootstrap support value that was decided to be definitive for subtype origin of each fragment. The x-axes in both illustrations signify the loci with respect to the HXB2 genome. SimPlot v3.5.1 software was used to conduct the similarity plot and bootscan analyses, where a sliding window of 400 nucleotides, overlapped by 40 nucleotides, with 1000 bootstrap replicates were adopted as the optimal parameters [4]. (**B**) The illustration on the right side of the figure displays the subregion confirmatory neighbor-joining tree analyses. MEGA X software was used to construct neighbor-joining trees for each of the fragments as defined by the prior similarity plot and bootscan analyses [1]. Kimura two-parameter nucleotide substitution model with 1000 bootstrap replicates, which was used to evaluate the reliability of the phylogenetic clustering, were adopted as the optimal parameters for the phylogenetic analyses [5]. Threshold of 70% bootstrap support value was decided to be definitive for subtype origin of each fragment. The loci of the beginning and end of each fragment in accordance with the HXB2 numbering are indicated above each respective phylogenetic tree. The branch displayed by a black intermittent line ending in a black dot demonstrates the query sequence. The color coding of the phylogenetic trees agrees with rest of the recombination analyses within the figure.

Sample BS72_KR017779 (Cameroon)

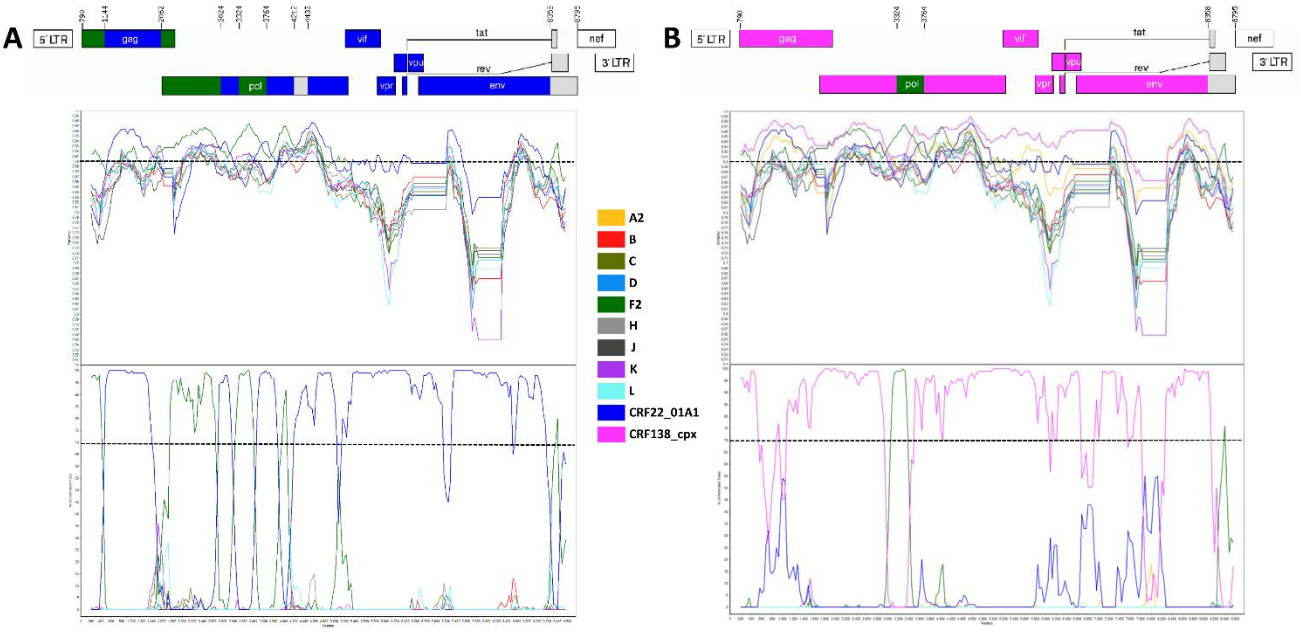

Figure S5: Recombination analyses of isolate BS72 (GenBank accession number: KR017779) using two different reference datasets. Isolate BS72 that was sampled in Cameroon, which was identified through BLAST analyses, demonstrate a very similar yet slightly different mosaic structure to the CRF138_cpx strain. The prior analyses of its intersubtype mosaic structure revealed that isolate BS72 is a unique recombinant form (URF) of the CRF138_cpx strain, “Rec. of 138_cpx, F2”. This comparative analysis was executed to clearly highlight the two slight differences between the mosaic genomic structures of isolate BS72 and CRF138_cpx strain, in order to emphasize the recombination events creating the URF of CRF138_cpx strain. MEGA X software was employed for the construction of the multiple sequence alignment of the query sequences and the reference dataset using the Clustal W algorithm [1,2]. AliView software was used to visualize and manually edit the multiple sequence alignment [3]. The finalized multiple sequence alignment was utilized for the following recombination analyses. The subtype origin of each fragment is color coded in agreement with all instructive recombination analyses, and the color coding is described in the middle of the figure. (**A**) The recombination analyses shown by the illustrations on the left side of the figure, were run against a reference dataset of HIV-1 group M subtypes (B, C, D, F2, H, J, K, and L) downloaded from the Los Alamos HIV Sequence Database (http://www.hiv.lanl.gov), which was enriched through Basic Local Alignment Search Tool (BLAST) analyses (https://www.hiv.lanl.gov/content/sequence/BASIC_BLAST/basic_blast.html) with six of the top BLAST hits for sub-subtype F2 and CRF22_01A1. The upper left illustration demonstrates the genomic map, as created using the Recombinant HIV-1 Drawing Tool (https://www.hiv.lanl.gov/content/sequence/DRAW_CRF/recom_mapper.html). The numbers above the illustration denote the intersubtype recombination breakpoints with respect to the HXB2 genome. The illustration shows the intersubtype mosaic pattern of the URF of CRF138_cpx strain, “Rec. of 138_cpx, F2”, in which the near full-length HIV-1 genome was divided into nine fragments based on the eight recombination breakpoints. The middle left illustration presents the similarity plot analysis,

where the y-axis shows the percent similarity of the query sequence to the reference dataset. The intermittent horizontal line on the similarity plot depicts the 90% similarity. The lower left illustration presents the bootscan analysis, in which the y-axis shows the bootstrap support value. The intermittent horizontal line on the bootscan depicts the threshold of 70% bootstrap support value that was decided to be definitive for subtype origin of each fragment. The x-axes in both illustrations signify the loci with respect to the HXB2 genome. SimPlot v3.5.1 software was used to conduct the similarity plot and bootscan analyses, where a sliding window of 400 nucleotides, overlapped by 40 nucleotides, with 1000 bootstrap replicates were adopted as the optimal parameters [4]. (**B**) The recombination analyses, shown by the illustrations on the right side of the figure, were run against a reference dataset of HIV-1 group M subtypes (A2, B, C, D, F2, H, J, K, and L) and CRF22_01A1 downloaded from the Los Alamos HIV Sequence Database (http://www.hiv.lanl.gov), which was enriched through Basic Local Alignment Search Tool (BLAST) analyses (https://www.hiv.lanl.gov/content/sequence/BASIC_BLAST/basic_blast.html) with one of the top BLAST hits for sub-subtype F2, along with the four samples from Cyprus belonging to the CRF138_cpx strain. The bootscan and similarity plot analyses were repeated using this reference dataset. The recombination analyses in part B show that there are two differences between isolate BS72 and CRF138_cpx strain at loci 3324-3761 and 8358-8795 (in the HXB2 genome).

References

1. Kumar, S.; Stecher, G.; Li, M.; Knyaz, C.; Tamura, K. MEGA X: Molecular Evolutionary Genetics Analysis across Computing Platforms. *Mol. Biol. Evol.* **2018**, *35*, 1547–1549, doi:10.1093/molbev/msy096.

2. Thompson, J.D.; Higgins, D.G.; Gibson, T.J. CLUSTAL W: improving the sensitivity of progressive multiple sequence alignment through sequence weighting, position-specific gap penalties and weight matrix choice. *Nucleic Acids Res.* **1994**, *22*, 4673–4680, doi:10.1093/nar/22.22.4673.

3. Larsson, A. AliView: a fast and lightweight alignment viewer and editor for large datasets. *Bioinformatics* **2014**, *30*, 3276–3278, doi:10.1093/bioinformatics/btu531.

4. Lole, K.S.; Bollinger, R.C.; Paranjape, R.S.; Gadkari, D.; Kulkarni, S.S.; Novak, N.G.; Ingersoll, R.; Sheppard, H.W.; Ray, S.C. Full-length human immunodeficiency virus type 1 genomes from subtype C-infected seroconverters in India, with evidence of intersubtype recombination. *J. Virol.* **1999**, *73*, 152–160, doi:10.1128/JVI.73.1.152-160.1999.

5. Kimura, M. A simple method for estimating evolutionary rates of base substitutions through comparative studies of nucleotide sequences. *J. Mol. Evol.* **1980**, *16*, 111–120, doi:10.1007/BF01731581.

6. Tavaré, S. Some probabilistic and statistical problems in the analysis of DNA sequences. *Lect Math Life Sci (Am Math Soc)* **1986**, *17*, 57–86.

7. Ragonnet-Cronin, M.; Hodcroft, E.; Hué, S.; Fearnhill, E.; Delpech, V.; Brown, A.J.L.; Lycett, S. Automated analysis of phylogenetic clusters. *BMC Bioinformatics* **2013**, *14*, 317, doi:10.1186/1471-2105-14-317.

8. Pineda-Peña, A.-C.; Theys, K.; Stylianou, D.C.; Demetriades, I.; Abecasis, A.B.; Kostrikis, L.G. HIV-1 Infection in Cyprus, the Eastern Mediterranean European Frontier: A Densely Sampled Transmission Dynamics Analysis from 1986 to 2012. *Sci. Rep.* **2018**, *8*, 1702, doi:10.1038/s41598-017-19080-5.

9. Pineda-Peña, A.-C.; Faria, N.R.; Imbrechts, S.; Libin, P.; Abecasis, A.B.; Deforche, K.; Gómez-López, A.; Camacho, R.J.; de Oliveira, T.; Vandamme, A.-M. Automated subtyping of HIV-1 genetic sequences for clinical and surveillance purposes: Performance evaluation of the new REGA version 3 and seven other tools. *Infect. Genet. Evol.* **2013**, *19*, 337–348, doi:10.1016/j.meegid.2013.04.032.
